# Hepatitis B Virus Infection and Immunopathogenesis in a Humanized Mouse Model: Induction of Human-Specific Liver Fibrosis and M2-Like Macrophages

**DOI:** 10.1371/journal.ppat.1004032

**Published:** 2014-03-20

**Authors:** Moses T. Bility, Liang Cheng, Zheng Zhang, Yan Luan, Feng Li, Liqun Chi, Liguo Zhang, Zhengkun Tu, Yanhang Gao, Yangxin Fu, Junqi Niu, Fusheng Wang, Lishan Su

**Affiliations:** 1 Lineberger Comprehensive Cancer Center, Department of Microbiology and Immunology, University of North Carolina at Chapel Hill, Chapel Hill, North Carolina, United States of America; 2 Center of Infectious Disease, Beijing 302 Hospital, Beijing, China; 3 Center for Infection and Immunity, Institute of Biophysics, Chinese Academy of Sciences, Beijing, China; 4 Department of Pathology, University of Chicago, Chicago, Illinois, United States of America; 5 Department of Translational Medicine, Department of Surgery, Department of Medicine, the First Hospital, Jilin University, Changchun, Jilin, China; Nationwide Children's Hospital, United States of America

## Abstract

The mechanisms of chronic HBV infection and immunopathogenesis are poorly understood due to a lack of a robust small animal model. Here we report the development of a humanized mouse model with both human immune system and human liver cells by reconstituting the immunodeficient A2/NSG (NOD.Cg-*Prkdc^scid^ Il2rg^tm1Wjl^*/SzJ mice with human HLA-A2 transgene) with human hematopoietic stem cells and liver progenitor cells (A2/NSG-hu HSC/Hep mice). The A2/NSG-hu HSC/Hep mouse supported HBV infection and approximately 75% of HBV infected mice established persistent infection for at least 4 months. We detected human immune responses, albeit impaired in the liver, chronic liver inflammation and liver fibrosis in infected animals. An HBV neutralizing antibody efficiently inhibited HBV infection and associated liver diseases in humanized mice. In addition, we found that the HBV mediated liver disease was associated with high level of infiltrated human macrophages with M2-like activation phenotype. Importantly, similar M2-like macrophage accumulation was confirmed in chronic hepatitis B patients with liver diseases. Furthermore, gene expression analysis showed that induction of M2-like macrophage in the liver is associated with accelerated liver fibrosis and necrosis in patients with acute HBV-induced liver failure. Lastly, we demonstrate that HBV promotes M2-like activation in both M1 and M2 macrophages in cell culture studies. Our study demonstrates that the A2/NSG-hu HSC/Hep mouse model is valuable in studying HBV infection, human immune responses and associated liver diseases. Furthermore, results from this study suggest a critical role for macrophage polarization in hepatitis B virus-induced immune impairment and liver pathology.

## Author Summary

Over 350 million people worldwide are chronically infected with the hepatitis B virus (HBV), which leads to severe liver diseases including fibrosis and cancer. The mechanisms of chronic HBV infection and disease are poorly understood due to a lack of a robust small animal model. Here we report a novel animal model that can be efficiently repopulated with both human immune and liver cells. The A2/NSG-hu HSC/Hep humanized mouse model supported persistent HBV infection, human immune responses, albeit impaired in the liver, chronic liver inflammation and liver fibrosis. In addition, we found that the HBV mediated liver immune impairment and liver disease was associated with high level of infiltrated human immuno-suppressive/pro-fibrogenic macrophages; this result was confirmed in chronic HBV-induced liver disease patients and acute HBV – induced liver failure patients. Importantly, we demonstrate that HBV promotes immuno-suppressive/pro-fibrogenic macrophage polarization in human macrophages using cell culture models. The humanized mouse model is a valuable platform in studying HBV infection, human immune response and liver diseases. Furthermore, results from this study suggest a critical role for macrophage activation in hepatitis B induced liver diseases, thus providing a novel therapeutic target.

## Introduction

Chronic hepatitis B virus (HBV) infection results in liver fibrosis/cirrhosis and development of hepatocellular carcinoma (HCC) [1], [2]. Establishment of chronic HBV infection is inversely associated with patient's age with neonatal and infants most susceptible, while adults are mostly resistant to chronic infection [3], [4]. Chronic HBV infection is associated with impaired immune responses to viral antigens and chronic inflammation in the liver, leading to progressive liver diseases. Though HBV-induced liver disease is predominately a chronic disease, requiring decades of chronic infection and liver inflammation [5], [6], [7], HBV infection occasionally results in accelerated liver disease and liver failure during acute infection [8], [9]. The development of preventive vaccines and therapeutics using chimpanzees and surrogate hepatitis virus-small animal models has played a significant role in preventing new infections and controlling HBV-induced liver diseases. However, HBV is endemic in many developing countries with over 350 million people worldwide chronically infected [10]. Delineation of the mechanisms by which HBV evades host immunity to establish chronic infection and promote liver disease is hampered by the lack of robust animal models [11], [12], [13].

HBV and other human hepatotropic pathogens including HCV have host species restriction, namely humans and chimpanzees. To overcome host species restriction barrier for *in vivo* infection and disease modeling, several human-murine chimeric liver models have been developed [14]. The Alb-uPA/SCID humanized mouse with high human adult hepatocyte repopulation can be infected with HCV/HBV [14]. Additionally, the fumarylacetoacetate hydrolase (Fah)-Rag2-γC-null mice also allow human hepatocytes engraftment and HCV infection [15], [16], [17]. However, these human-murine chimeric liver models lack a functional human immune system, thus it is not possible to study host immune response and hepatitis virus-induced immunopathology [14], [17]. To overcome the limitations associated with current chimeric human-murine liver mouse models, we have recently developed a humanized mouse model with both human immune system and liver cells (AFC8-hu HSC/Hep mice) [18], [19]. AFC8-hu HSC/Hep mice can support HCV infection in the liver and generate human T-cell response to HCV. Additionally, HCV infection induces liver inflammation and fibrosis, correlated with activation of human hepatic stellate cells and expression of human fibrogenic genes [18].

Chronic liver inflammation and associated pathology in chronic HBV infection is characterized by infiltration of various leukocyte populations including activated macrophages. Several reports suggest that HBV promotes macrophage activation and M2 polarization [20], [21], [22]. Macrophages play a critical role in modulating pathogen clearance, chronic inflammation and associated liver pathology; with M1 polarized macrophages promoting pathogen clearance, and M2-like polarized macrophages impairing host immunity and promoting tissue fibrosis/remodeling [23], [24], [25], [26], [27].

In this study, we developed a humanized mouse model by injecting human liver progenitor cells (Hep) and CD34+ human hematopoietic stem cells (HSC) directly into the liver of newborn A2/NSG (HLA-A2 transgenic NOD *scid* IL2 receptor gamma chain knockout mice [28], [29], [30]). The A2/NSG mouse lacks NK cells and T/B-lymphocytes. They support efficient development of a functional human immune system after injecting CD34+ human hematopoietic stem cells (HSC) into the liver of newborn mice [30], [31]. Furthermore, the A2/NSG mouse carries the human HLA-A2 transgene, which enhances development of human MHC-restrict T lymphocytes [30]. To promote human liver cell repopulation, A2/NSG-hu HSC/Hep mice were treated with a murine specific anti-Fas agonistic antibody (Jo2) [32], [33], [34], [35], [36]. The A2/NSG-hu HSC/Hep mouse model enabled human liver and immune system development and supported long-term HBV infection, anti-HBV human immune response and HBV-induced liver diseases including hepatitis and fibrosis. Interestingly, we also observed accumulation of activated human M2-like macrophages in the HBV-infected humanized liver. Importantly, similar M2-like macrophage accumulation was confirmed in chronic HBV patients and HBV-induced acute liver failure patients. Importantly, inoculation of human macrophages culture with HBV positive supernatant resulted in M2–like activation.

## Results

### The A2/NSG-hu HSC/Hep mouse model supports persistent HBV infection

**Figure 1:**
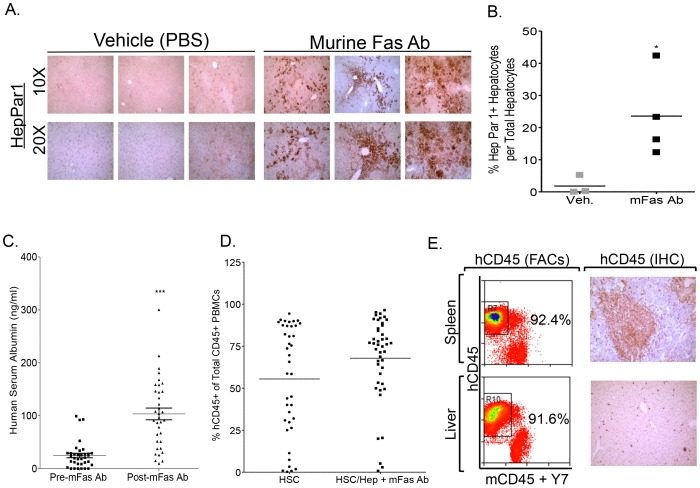
Murine-specific Fas antibody treatment promotes human liver reconstitution in A2/NSG-hu HSC/Hep mice. **A–B:** Anti-mouse Fas (mFas) antibody enhances human hepatocyte repopulation in A2/NSG immunodeficient mice transplanted with human HSC and liver progenitor cells. Littermate A2/NSG mice transplanted with human CD34+ HSC and hepatocyte progenitors (HSC/Hep) were treated with vehicle or mFas activating antibody and sacrifice at 3 months post transplantation. (**A**) Liver sections from vehicle (PBS) or Fas antibody treated humanized mice were stained with anti-human HepPar1 monoclonal antibody. (**B**) % Hep Par1+ cells per total liver cells for each mouse was quantified using 5 different fields and summarized. *, p<0.05. (**C**) Elevated human serum albumin levels in Jo2 (mFas Ab) treated A2/NSG-hu HSC/Hep mice compared to pre-Jo2 (mFas Ab) treatment (n = 35, 3 cohorts). (**D**) Comparative analysis of human immune reconstitution (hCD45%) in A2/NSG/Fas-hu HSC/Hep mice and A2/NSG-hu HSC mice (n = 40, 3 cohorts). (**E**) Human immune reconstitution of liver and lymphoid tissue (spleen) in A2/NSG/Fas-hu HSC/Hep mice. Total leukocytes from indicated tissues were stained with human (hCD45+) and murine (mCD45+) leukocyte antibody plus dead cell marker (Y7).

We utilized the murine Fas activating antibody (Jo2 antibody) to induce murine-specific hepatocytes death in order to promote human hepatocytes repopulation. We confirmed the specie-specificity of Jo2 antibody [32] by incubating human liver cell line (HepG2) with Jo2 antibody. Jo2 antibody did not stain the human hCD95+ hepatocyte cell line (Figure S1A). Furthermore, human fetal liver progenitor cells were resistant to Jo2 antibody - mediated apoptosis, while A2/NSG mice were susceptible to Jo2 - induced liver damage (Figure S1B, S1C). Jo2 antibody treatment of mice transplanted with CD34+ HSCs and liver progenitor cells resulted in a significant increase in Hep Par1 positive human hepatocytes compared to vehicle treated animals at approximately 3 months post transplantation (Figure 1A, 1B). No significant liver disease was observed in Jo2 antibody treated animals at termination, thus confirming that low dose Jo2 mediated liver damage is transient and does not induce long-term liver damage (Figure 1A). Human serum Albumin levels were significantly elevated in Jo2 antibody treated transplanted animals compared to vehicle treated animals at 3 months post transplantation (Figure 1C). Additionally, Jo2 antibody treated A2/NSG animals transplanted with CD34+ HSCs and liver progenitor cells supported robust human immune cells repopulation (∼75% PBMCs are human CD45^+^), which was comparable to A2/NSG animals transplanted with HSCs only and not treated with Jo2 (Figure 1D). Human immune reconstitution resulted in the repopulation of blood, lymphoid tissues and liver with human leukocytes (hCD45+) including T cells (hCD45+ hCD3+), B cells (hCD45+ hCD19+), monocytes/macrophages (hCD45+ hCD3− hCD19− hCD56− hHLADR+ hCD14^high^ hCD11c^high^), myeloid dendritic cells (hCD45+ hCD3− hCD19− hCD56− hHLADR+ hCD14^low^ hCD11c^high^) and plasmacytoid dendritic cells (hCD45+ hCD3− hCD19− hCD56− hHLADR+ hCD123^high^ hCD4^high^) (Figure 1E, Figure S2).

**Figure 2:**
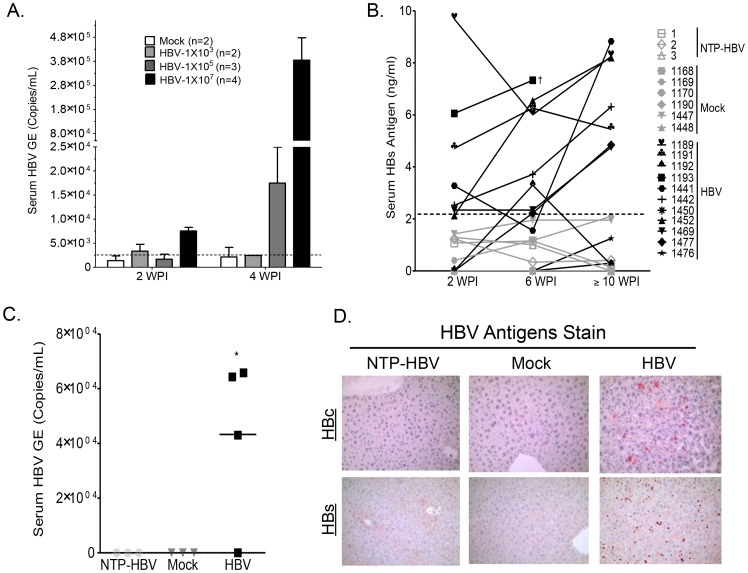
Persistent HBV infection in A2/NSG/Fas-hu HSC/Hep mice. (**A**) A2/NSG/Fas-hu mice or non-humanized mice were inoculated with PBS or HBV (1×10e3, 10e5 or 10e7 GE/mouse). Blood samples were collected at various times after infection. HBV genomic DNA was examined in sera from humanized mice infected with HBV at indicated titration dose and time points. (**B**) A2/NSG/Fas-hu mice or non-humanized mice were inoculated with PBS or HBV (1×10e6 GE/mouse). Blood samples were collected at various times after infection and HBs antigen in sera was measured by ELISA. (**C**) HBV genomic DNA was detected in sera from HBV-infected humanized mice at termination time points (14–16 wpi). †: Unable to bleed animal at later time points. (**D**) Liver samples were collected at termination time points (12–16 wpi). HBV core and surface antigens were detected in livers of HBV inoculated humanized mice and not in control groups.

To determine if the A2/NSG-hu HSC/Hep mouse can support HBV infection, humanized and non-humanized mice were inoculated with HBV patient isolates at 1×10e3, 10e5 or 10e7 genome copies per mouse (Figure 2A). HBV viral infection was examined by measuring serum levels of HBV genome and HBsAg. At 10e5 HBV genome copies/mouse, HBV replication was detected at 4 weeks post-infection (wpi), whereas low levels of HBV genomes were detected at 2 wpi (10e4 copies/ml) and reached higher levels at 4 wpi (4×10e5 copies/ml) in mice inoculated at 10e7 HBV/mouse (Fig. 2A). Serum HBs antigen was persistently detected in approximately 75% of HBV inoculated humanized mice (HBV), but not in non-transplanted control mice inoculated with HBV (NTP-HBV) or mock inoculated humanized mice (Mock) (Figure 2B, Table S1). Additionally, serum HBV genome was detected in approximately 75% of HBV inoculated humanized mice but not in HBV inoculated non-humanized mice or mock inoculated humanized mice at sacrifice time point (Figure 2C). To examine HBV infection in the liver, animals were sacrificed at approximately 12–16 weeks post inoculation. HBV core and surface antigens were detected in the livers of all humanized mice with detectable HBV viremia, but not in the livers of control animals (non-transplanted mice inoculated with HBV and mock inoculated humanized mice) (Figure 2D, Table S1). Additionally, HBV genomes were detected in HBV infected A2/NSG/Fas-hu HSC/Hep livers (Figure S3).

### HBV infection induces human immune response in the A2/NSG/Fas-hu HSC/Hep mouse

**Figure 3:**
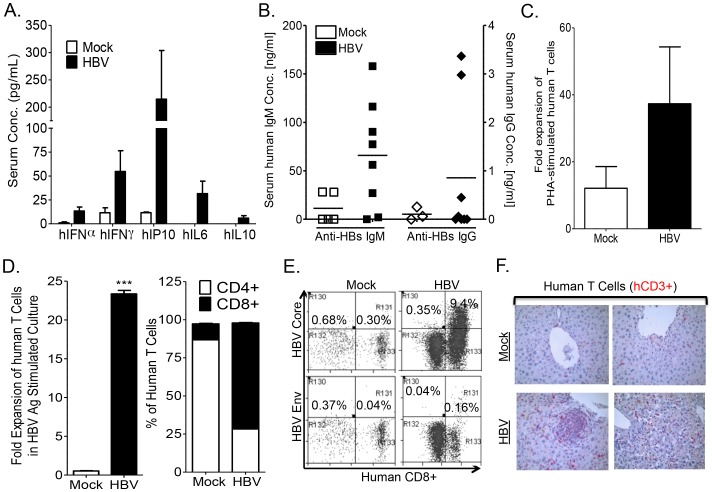
HBV infection induces anti-HBV human immune response in A2/NSG/Fas-hu mice. HBV infection results in human immune response with induction of serum levels of human inflammatory cytokines (**A**), B cells response (serum IgM and IgG antibodies levels) (**B**) and T cell response (**C–F**). (**C–F**): Expansion of human T cells following stimulation with PHA or HBV antigens plus anti-CD28 mAb (14 days with IL7 and IL2) of human lymphoid tissue T cells from mock and HBV infected mice. Total human T cell expansion for PHA (**C**), HBV antigen and resulting percentage of expanded CD4+ and CD8+ T cells (**D**) following stimulation are presented. Error bars are shown as standard deviations. (**E**) HBV infection induced HLA-A2 restricted HBV-core (18–27)- or HBV-envelope (183–191)-specific CD8+ T cells. Antigen specific CD8+ T cells were detected after expansion with HBV antigens for 14 days as above. The immunodominant HBV-core (18–27) epitope induced higher levels of CD8+ T cells than the HBV-envelope (183–191) epitope. Spleen cells from mock-infected mice did not respond to stimulation with HBV antigens and showed no detectable antigen-specific T cells. (**F**) HBV infection and associated immune response induced liver infiltration of human T cells. Liver sections from Mock and HBV inoculated humanized mice sacrificed at 12–16 weeks post inoculation were stained with human CD3 (human T cells, red) antibodies. No significant leukocyte infiltration was observed in livers from mock animals.

To characterize human immune response to HBV infection in humanized mice, human cytokines, B cell and T cells responses were examined. Elevated levels of human cytokines were detected in the serum of HBV infected mice with relatively high levels of IFNγ, IP10 and IL6, along with low levels of IL10 and IFNα (Figure 3A). Additionally, HBV infection also resulted in anti-HBV humoral response. We detected elevated levels of anti-HBs IgM antibody in infected humanized mice compared to control animals (Figure 3B). However, antigen-specific IgG response was detected in only two of eight mice and at very low levels as reported in other human B cell studies with humanized mice [37]. To characterize anti-HBV human T cell immune response, HLA-A2 donor derived-leukocytes from the spleen and lymph nodes of mock- or HBV-infected humanized animals were collected and stimulated *in vitro* with PHA or A2-restricted HBV peptides plus anti-CD28 mAb and expanded in the presence of IL2 and IL7 for 2 weeks. Human T cells from both mock and HBV infected humanized mice responded to PHA stimulation, however higher expansion of T cells from HBV infected mice was detected than T cells from mock controls (Figure 3C). Analysis of human T cell expansion following HBV antigen stimulation showed robust expansion (∼20 fold) of human T cells from HBV-infected humanized mice and no expansion of human T cells from mock controls (Figure 3D). Additionally, stimulation with the A2-restricted peptides preferentially expanded CD8+ T cells (Figure 3D). A2/HBV peptide pentamer staining showed significantly higher frequency of the immunodominant HBV Core peptide (aa18–aa27) specific human CD8^+^ T cells (Figure 3E). Similarly as in human studies, HBV Core (aa18–aa27) pentamer+ CD8+ T cells exhibited significantly higher frequency than the HBV Env (aa183–aa191) pentamer+ CD8+ T cells (Figure 3E). As expected, infiltration of human T cells was detected in the liver of HBV infected humanized mice compared to mock animals (Figure 3F).

### Chronic HBV infection induces chronic hepatitis and human liver fibrosis in A2/NSG/Fas-hu mice

**Figure 4:**
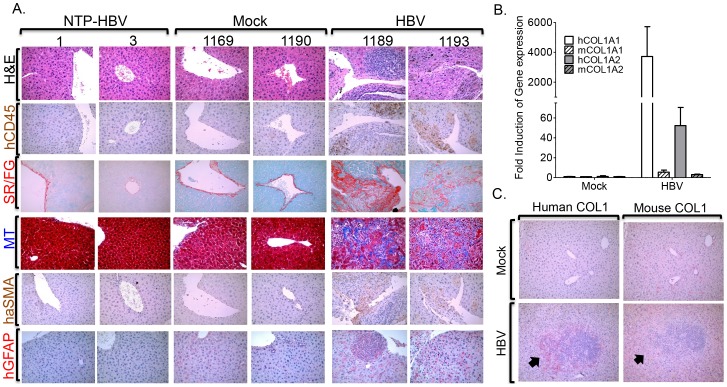
HBV infection induces chronic hepatitis and human liver fibrosis. A2/NSG/Fas-hu HSC/Hep mice were inoculated with Mock or HBV and terminated at 12–16 weeks post infection. (**A**) Representative liver sections from sacrificed HBV infected or control mice stained with H&E and hCD45 to examine human leukocyte infiltration and Sirius red/fast green (SR/FG) and Masson's trichrome (MT) stains to examine liver fibrosis. Human specific α-SMA (alpha-smooth muscle actin) and GFAP (glial fibrillary acidic protein) antibodies were used to detect activated human hepatic stellate cell activation or myofibroblasts. Livers from two representative mice per group are shown. Gene expression analysis of human and mouse collagen 1 was examined using species-specific primers (**B**) and antibodies (**C**). A black arrow denotes marking indicating same region.

Chronic HBV infection in patients is associated with chronic hepatitis and liver fibrosis, characterized by leukocyte infiltration and collagen deposition in portal/periportal regions of the liver [38]. To examine leukocyte infiltration and fibrosis in HBV infected liver of humanized mice, liver sections were examined at time of sacrifice. HBV infection resulted in significant infiltration of human leukocytes (blue cells - H&E, brown cells - hCD45+) in the portal/periportal regions of infected livers (Figure 4A). Hepatitis was absent in control animals (mock inoculated humanized mice and non-transplanted mice inoculated with HBV) (Figure 4A).

Liver fibrosis is characterized by activation of hepatic stellate cells, which promote increased collagen deposition that results in tissue pathology [39]. Gross morphological examination of livers from HBV infected humanized animals also showed extensive tissue scarring; control livers were morphologically normal (Figure S4). Examination of liver fibrosis using Sirius red/fast green (SR/FG) and Masson's trichrome (MT) staining showed increased collagen deposition in livers of HBV infected humanized mice but not of control animals (Figure 4A, Table S1). HBV-induced liver fibrosis was associated with elevated activation/expansion of human hepatic stellate cells/myofibroblasts as measured by human αSMA- and GFAP-positive cells (Figure 4A). Additionally, human collagen 1 expression but not mouse collagen 1 expression was unregulated in HBV-induced fibrotic liver tissues (Figure 4B, 4C).

### Anti-HBs neutralizing antibody treatment prevents HBV infection and associated liver diseases in the A2/NSG/Fas-hu mouse model

**Figure 5:**
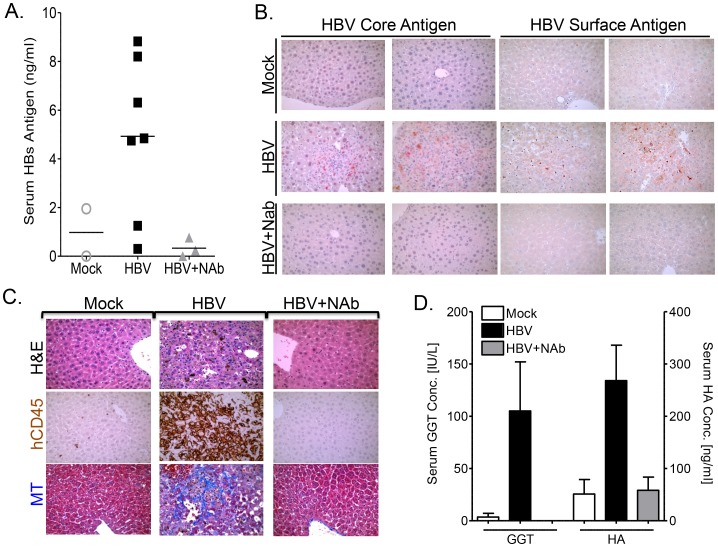
Anti-HBs neutralizing antibody prevents HBV infection and associated liver diseases. A2/NSG/Fas-hu HSC/Hep mice were inoculated with HBV +/− anti-HBs neutralizing antibody (NAb) terminated at 10–16 week post infection. (**A**) Serum level of HBs antigen was measured at sacrifice time point in mock, HBV alone or HBV + anti-HBs antibody groups. (**B**) Liver sections from mock, HBV or HBV plus anti-HBs antibody treated animals (representative two mice per group) were stained for HBV core or surface antigens. (**C**) Representative liver sections from mock, HBV or HBV plus anti-HBs antibody treated animals were stained with H&E and hCD45 to examine human leukocyte infiltration and Masson's trichrome (MT) stains to examine liver fibrosis. (**D**) Liver fibrosis was also examined using serum biomarkers (GGT and HA) in mock (n = 6), HBV (n = 7) or HBV plus anti-HBs antibody treated (n = 3) animals.

To demonstrate that HBV is the pathogenic agent in human HBV+ serum-induced liver disease in the A2/NSG/Fas-hu mouse model, humanized animals were inoculated with HBV in the presence of anti-HBsAg neutralizing antibody (NAb) (Table S1). HBs antigens in the blood were detected in ∼75% of humanized animals inoculated with HBV alone but not in anti-HBsAg neutralizing antibody treated or control groups (Figure 5A, Table S1). Immunohistochemical analysis showed the presence of HBV core and surface antigens in the liver of HBV-infected, but not in the HBV-neutralizing antibody or mock inoculated mice (Figure 5B, Table S1). As expected, anti-HBs neutralizing antibody treatment also blocked HBV-induced liver diseases, including chronic hepatitis (leukocytes, hCD45+ cells) and liver fibrosis (MT) (Figure 5C, Table S1). Additionally, elevated levels of serum biomarkers of liver fibrosis (gamma-glutamyl transpeptidase - GGT and hyaluronic acid - HA) were detected in HBV infected humanized mice compared to mock animals; neutralizing antibody treatment blocked the induction of these serum biomarkers (Figure 5D).

### Persistent HBV infection is associated with impaired anti-HBV human immune response in the liver

**Figure 6:**
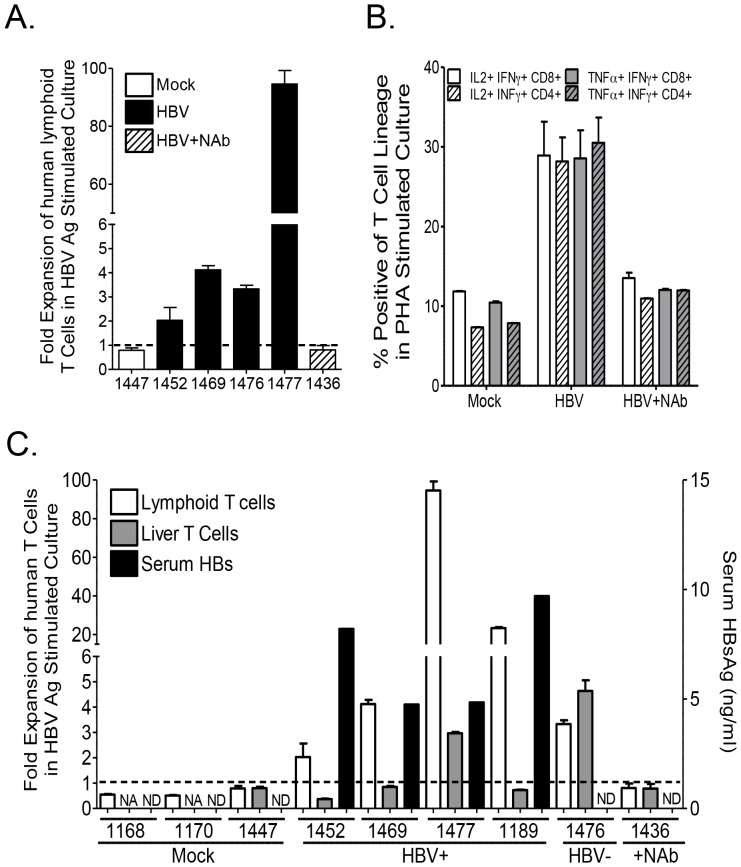
Persistent HBV infection is associated with liver specific immune impairment. Expansion of human T cells following stimulation with PHA or HBV antigens plus anti-CD28 mAb (14 days with IL7 and IL2) of human T cells from liver or lymphoid tissues of mock, HBV plus neutralizing antibody or HBV infected mice. (**A**) Total human T cell expansion after 2 weeks of stimulation with HBV antigen was examined. (**B**) Th1 associated double positive cytokine production in PHA expanded T cells re-stimulated with PMA plus ionomycin was also examined. (**C**) Comparative analysis of HBsAg level in individual animals (Mock, PBS inoculated; HBV+, HBV inoculated with persistent HBV infection; HBV−, HBV inoculated with no infection; +NAb, HBV plus anti-HBsAg neutralizing antibody inoculated), and associated human liver and lymphoid tissue T cell expansion following HBV antigen stimulation. NA (Not applicable, indicating animals with low number of T cells in the liver below the assay requirements, thus not tested). ND (Not detectable).

Several studies have suggested liver specific T cell immune impairment in chronic HBV infection [5]. To characterize anti-HBV human T cell immune response in lymphoid and liver tissues, HLA-A2 donor derived-leukocytes from the spleen and lymph nodes or livers of mock-, HBV plus neutralizing antibody- or HBV-infected humanized animals were collected and stimulated/expanded *in vitro* with PHA or A2-restricted HBV peptides plus anti-CD28 mAb and expanded in the presence of IL2 and IL7 for 2 weeks. Analysis of human T cell expansion following HBV antigen stimulation showed robust expansion of human lymphoid T cells from HBV-infected humanized mice; lymphoid T cells from mock or HBV plus neutralizing antibody inoculated animals exhibited refractory response to HBV antigen stimulation (Figure 6A). Additionally, PMA plus ionomycin re-stimulation of PHA expanded human lymphoid T cells resulted in enhanced cytokine production (IFNγ/IL2 or IFNγ/TNFα double positive cells) by T cells from HBV infected animals compared to mock or HBV plus anti-HBs neutralizing antibody inoculated animals (Figure 6B; Figure S5). Comparative analysis of liver and lymphoid tissue T cell expansion following HBV antigen stimulation showed robust expansion of lymphoid T cells from HBV-infected humanized mice; however, liver T cells exhibited significantly lower response compared to lymphoid T cells from the same persistently infected animal with the exception of an HBV inoculated animal (#1476) that did not developed persistent infection (Figure 6C). Both lymphoid and liver T cells from mock and HBV plus neutralizing antibody inoculated animals exhibited no significant response to HBV antigen stimulation (Figure 6C).

### HBV-induced liver disease and immune impairment in the liver is associated with induction of M2-like macrophages

**Figure 7:**
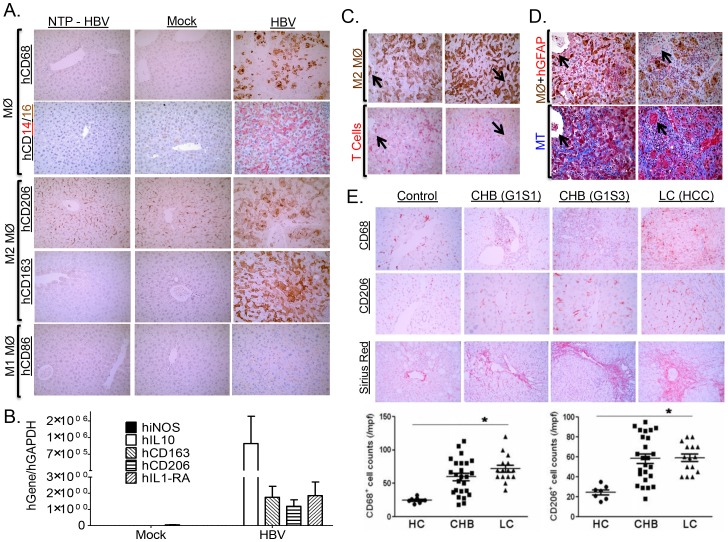
HBV-induced liver disease and immune impairment is associated with M2-like macrophage activation. A2/NSG/Fas-hu HSC/Hep mice were infected with Mock or HBV and terminated at 12–16 weeks post infection. (**A**) Infiltrating monocyte/macrophage (CD68+, CD14^high^, CD16^low/medium^) from HBV-infected and control livers were stained for M1-like marker (CD86+) and M2-like markers (CD163+, CD206+). (**B**) Elevated levels of human M2-like macrophage gene expression profile in HBV infected humanized livers. (**C–D**) HBV-induced M2-like macrophage co-localized with human T cells (**C**), activated hepatic stellate cells and fibrotic regions (**D**) in infected humanized livers. (**E**) Sex and age matched control and chronic HBV patients at varying stages of liver diseases were examined to characterize macrophages (CD68+) in chronic HBV infection. Representative M2-like marker (CD206+) and fibrosis (Sirius red) were stained in healthy controls and chronic HBV-infected human livers. CHB, Chronic HBV infection; G1S1/G1S3, stage 1/stage 3; LC, chronic HBV associated hepatocyte cell carcinoma. Chronic HBV-induced fibrotic (CHB) and liver cancer (LC) patients exhibited elevated levels of macrophages (CD68+) of M2-like lineage (CD206+) in the liver. A black arrow denotes marking indicating same region.

Several studies have indicated that macrophages play a critical role in modulating pathogen clearance, chronic inflammation and associated tissue pathology; with M1-like macrophages promoting pathogen clearance, and M2-like macrophages impairing Th1 immune response and promoting tissue fibrosis/remodeling/wound healing [40]. Immunohistochemical analysis of HBV associated liver inflammation in humanized mice showed high levels of human macrophages with predominately “M2-like” phenotype (hCD68^high^, hCD14^high^, hCD16^low/medium^, hCD163^high^, hCD206^high^, hCD86^negative^) (Figure 7A). Additionally, gene expression analysis also confirms elevated levels of human M2 macrophages (hiNOS^negative^, hIL10^high^, hCD163^high^, hCD206^high^, hIL1RA^high^) (Figure 7B). Several studies have demonstrated that M2-like macrophages are potent immune suppressor cells expressing high levels of IL10, co-inhibitory molecules (B7-H4), while depleting L-arginine and down-regulating IL12, TNFα and co-stimulatory molecules (CD86); all factors critical for Th1 anti-viral immune response [41]. Analysis of liver inflammation in HBV infected humanized mice showed human M2 macrophages co-localized with human T cells (Figure 7C, Table S2). Additionally, liver analysis of HBV inoculated humanized animals that did not develop persistent infection (ID# 1450 and ID# 1476), showed the absence of M2-like macrophages was associated with the absence of persistent infection and associated liver disease (Figure 6C, Table S2). Interestingly, analysis of T cell response to antigen stimulation showed similar robust induction for both lymphoid and liver T cells (ID# 1476); this is in contrast to persistently infected animals that exhibited robust lymphoid T cell response but relatively refractory liver T cell response (Figure 6C, Table S2). Furthermore, analysis of Liver and spleen from the same persistently infected animals showed M2 macrophages preferentially localized to the liver and not the spleen; thus associating the presence of M2-like macrophages in the liver with T cell impairment (Figure S6). M2-like macrophages also localized with activated human hepatic stellate cells (hGFAP+) and fibrotic regions (blue regions) in HBV infected humanized livers (Figure 7D, Figure S7, Table S2). These results suggest that persistent HBV infection-induced liver disease and immune impairment in humanized mice is associated with M2-like macrophage infiltration.

To confirm our findings in human HBV patients, patient groups with varying degree of chronic HBV-induced liver diseases (Fibrosis/HCC) were examined. Analysis of chronic HBV associated liver diseases in humans showed high levels of predominately “M2-like” macrophage (CD68^high^, CD206^high^) in the liver infiltration (Figure 7E). Though chronic HBV-induced liver disease accounts for the vast majority of HBV associated morbidity/mortality, acute HBV infection occasionally results in accelerated liver disease and liver failure with subsequent mortality in the absence of liver transplantation [8]. Analysis of liver gene expression profile in acute HBV-induced liver failure patients also showed increased macrophage infiltration (CD68 upregulation), up-regulation of M2-like macrophage genes (IL10RA - Interleukin 10 receptor alpha subunit, Dectin-1, CD163, CD163L1, MRC1 (CD206) - C-type mannose receptor 1, MRC2 - C-type mannose receptor 2, AMAC1 - alternative macrophage activation-associated CC chemokine-1, IL10, B7-H4) and down-regulation or no change of M1-like macrophage genes (TNFα, iNOS, IL12p40) in HBV infected patients compared to healthy controls (Figure S8, S9) [8], [9]. Furthermore, liver gene expression profile analysis showed M2-like macrophage gene expression profile (CD68^high^, CD163^high^, AMAC1^high^, iNOS^low^, TNFα^low^) is associated with upregulation of tissue fibrosis (COL1A1^high^, TIMP1^high^) and damage markers (HMGB1^high^ [42], [43], [44], S100A9^high^ [45], [46]) (Figure S9) [8], [9].

**Figure 8:**
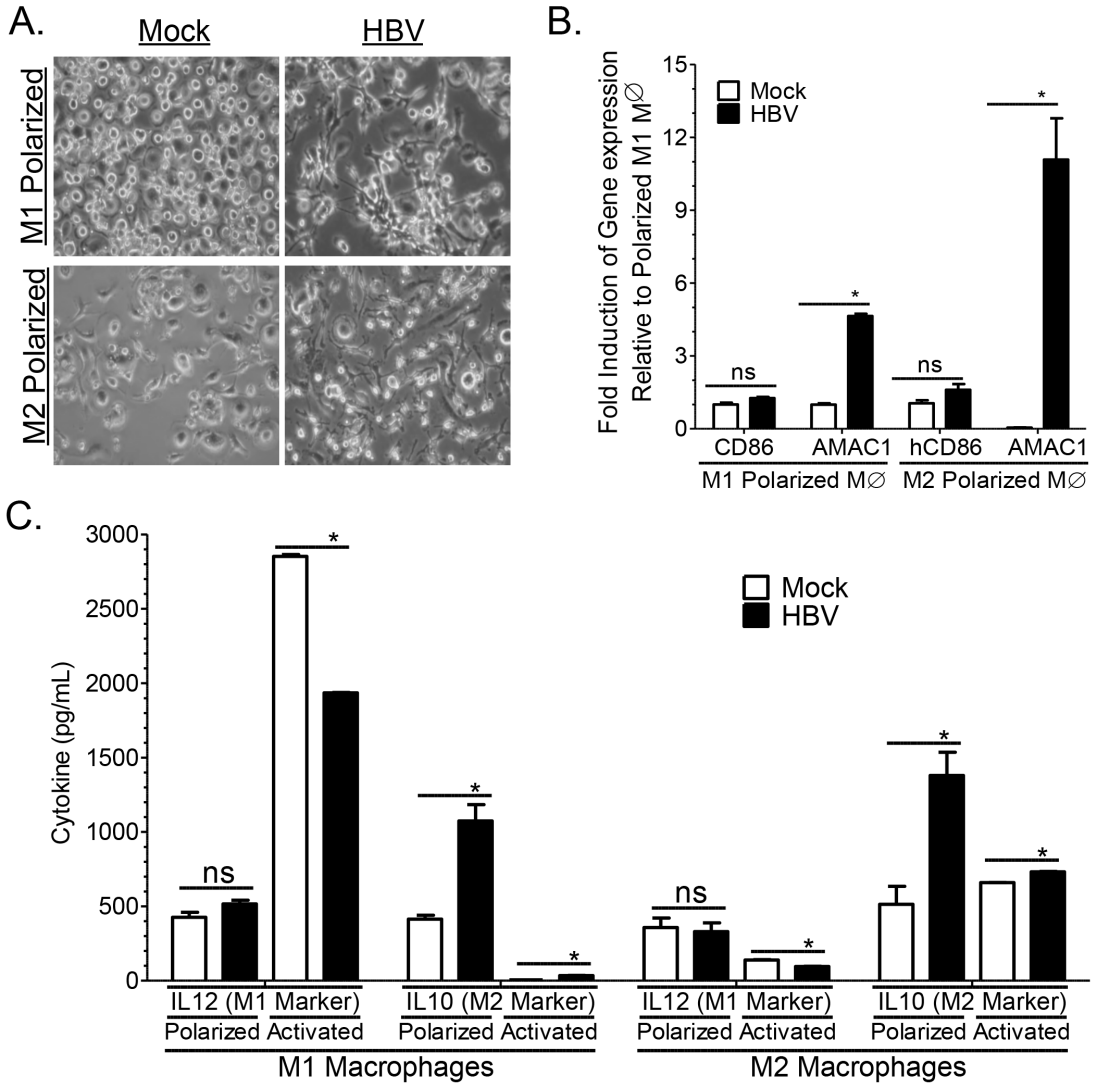
HBV induces M2-like activation in human macrophages. (**A–B**) Polarized M1 or M2 monocyte-derived macrophages were treated with HBV (HepG2.2.15 derived), or mock (HepG2 derived) for 6 days and M1 and M2 macrophage activation was examined using (**A**) morphological analysis (oval/round shape – M1, spindle/fibroblast shape – M2) and (**B**) gene expression analysis (CD86 – M1, AMAC1 – M2). (**C**) Polarized or activated (enhanced polarization) M1 or M2 monocyte-derieved macrophages were treated with HBV (HepG2.2.15 derived), or mock (HepG2 derived) for 6 days and M1 and M2 macrophage activation was examined using cytokine analysis (IL12 – M1, IL10 – M2).

Results from several cell culture studies have suggested that HBV can modulate monocyte activation resulting in induction of M2 associated cytokines and inhibition of M1 associated cytokines [20], [22], [47], [48], [49]. Here we demonstrate that HBV viral stock promoted M2-like macrophage activation in both human M1 and M2 polarized macrophages as examined by the induction of spindle/fibroblast shaped morphology (as opposed to round/oval shaped morphology of M1 macrophages) and M2 associated gene expression (AMAC1^high^, CD86^low^) (Figure 8A, 8B). Additionally, HBV inoculation resulted in induction of M2-like cytokine markers (IL10^high^ and IL12^low^) in both M1 and M2 polarized macrophages (Figure 8C). Activation of M1 and M2 polarized macrophages with activating cytokines results in enhanced polarization/activation of the respective lineage. Interestingly inoculation of M1 and M2 activated macrophages with HBV also resulted in the induction of IL10 and the inhibition of IL12 secretion (Figure 8C). Together these results suggest that HBV promotes M2-like macrophage activation to impair Th1 immune response and promote liver fibrosis/pathology.

## Discussion

We report here a humanized mouse model engrafted with both human immune cells and liver cells. The A2/NSG/Fas-hu HSC/Hep mouse supported persistent HBV infection, which induced human immune response, albeit impaired in the liver, chronic hepatitis and liver fibrosis. Therefore, the A2/NSG/Fas-hu mouse provides a novel humanized mouse model with both human immune and liver cells for studying hepatotropic pathogen infection and associated liver diseases. More importantly, this humanized mouse model strategy can be readily applied across current and future immunodeficient mouse models to promote both human liver and immune cells repopulation.

Immunodeficient mice expressing the uPA transgene in the liver of SCID mice or with mutant Fah genes allow transplanted human adult hepatocytes to have a growth advantage and efficiently repopulate the liver [50]. However, these mice have disadvantages including neonatal death, poor health and, most importantly, the lack of a human immune system [19]. To overcome these deficiencies, the A2/NSG/Fas-humanized mouse model enables inducible depletion of murine hepatocytes through the Fas apoptotic signaling pathway, resulting in elevated human liver repopulation in mice transplanted with human liver progenitor and hematopoietic stem cells [32], [33], [34], [36], [51]. Additionally, the A2/NSG background permits highly efficient engraftment and development of human xenografts including human hematopoietic stem cells compared to current immunodeficient mouse models [52]. Lower levels of human hepatocytes were detected in A2/NSG/Fas-humanized mice in comparison to the uPA or FAH mice transplanted with adult human hepatocytes. However, it should be noted that fetal liver cell repopulation is also low in those mouse models [53]. Genetic modification of human liver cells for enhanced survival, repopulation and differentiation coupled with mouse Fas agonist (Jo2) [34] and/or the AFC8 murine liver damage system [18] could further enhance human liver repopulation.

To examine the applicability of the A2/NSG/Fas-humanized mouse model for HBV infection studies, we inoculated humanized animals with clinical HBV isolates. We detected persistent HBV surface antigens and HBV genome in the sera of inoculated animals. Additionally, HBV core and surface antigens were detected in the livers of inoculated mice over 3–4 months after infection, which indicate that the A2/NSG/Fas-humanized mouse model supports persistent HBV infection. Importantly, treatment with anti-HBs neutralizing antibodies prevented HBV infection in the A2/NSG/Fas-hu mouse model. The A2/NSG/Fas-hu HSC/Hep mouse model enabled the development of human immune cells in the blood, lymphoid tissues and the liver, thus anti-HBV immune response was examined. Ex vivo T cell activation analysis showed that human lymphoid T cells from HBV infected humanized mice exhibited robust expansion in response to HBV antigen stimulation. Furthermore, elevated human anti-viral cytokines were detected in HBV infected humanized mice. Robust anti-HBV B cell response is very critical for vaccine associated prevention of HBV infection. However, only suboptimal B cell response has been reported thus far in humanized mouse models [52]. In concordance with those studies, we detected predominantly human IgM antibodies with anti-HBV activity in HBV infected animals. Although human lymphoid T cells exhibited robust anti-HBV immune responses, HBV infection resulted in persistent infection in approximately 75% of inoculated animals, which was associated with liver specific T cell impairment. The low HBV viremia in the blood of HBV-inoculated humanized mice may be due to the relatively low level of human hepatocyte engraftment (∼20%) and the immature human hepatocytes derived from fetal liver progenitor cells. Additionally, the anti-HBV immune response could also contribute to the low viremia.

In chronically infected patients, immune and inflammatory responses against HBV are implicated as the major mediators of liver diseases [54], [55]. Chronic HBV infection in the liver of A2/NSG/Fas-hu mice was associated with significant human leukocyte infiltration, leading to human hepatic stellate cell activation and human liver fibrosis. Several reports have shown macrophage activation/polarization plays a critical role in modulating pathogen clearance, chronic inflammation and associated tissue fibrosis and damage; with M1 polarized macrophages promoting anti-virus Th1 immune response and pathogen clearance, while M2 polarized macrophages impair Th1 immune response and promoting tissue remodeling [23], [24], [25], [26], [27]. M2 macrophages are critical innate immune cells involved in tissue remodeling/wound repair, secreting anti-inflammatory cytokines and redistributing micronutrients to sites of wound repair; however, during chronic infection, M2-like macrophages promote tissue fibrosis, neoplasia and impair Th1 response thus promoting pathogen persistence and associated tissue pathology [40]. We report that liver inflammation and immune impairment in chronic HBV infected humanized mice livers was associated with M2-like macrophages, which also localized to fibrotic regions. Most importantly, results from chronic HBV and acute HBV-induced liver disease/failure patients confirmed that accumulation of M2-like macrophages correlated with liver disease progression and failure. Several studies have reported that HBV virus/HBV-encoded proteins can directly promote M2-like activation [20], [21], [22]. We confirmed and extended those results by demonstrating that HBV promotes M2 macrophage polarization in human M1 and M2 macrophages.

In summary, we have established a novel humanized mouse model with both human liver cells and immune system (A2/NSG/Fas-hu HSC/Hep mice) using a highly immunodeficient mouse strain that efficiently supports human cell engraftment. A2/NSG/Fas-hu HSC/Hep mice were susceptible to chronic HBV infection, associated with HBV-specific human immune responses and liver immune impairment, chronic inflammation and fibrosis. Importantly, our findings suggest a critical role for M2-like macrophages in HBV infection, immune dysregulation and associated liver diseases.

## Materials and Methods

### Ethics statement

All animal experiments were conducted following NIH guidelines for housing and care of laboratory animals and in accordance with The University of North Carolina at Chapel Hill in accordance with protocols approved by the institution's Institutional Animal Care and Use Committee (protocol number 10-107). Human study protocols were approved by Beijing 302 Hospital Research and Ethics Committee and the independent ethics committee (IEC) of Jilin University; written informed consent was obtained from all participants. Liver gene expression profile analyses in patients were obtained from a dataset in Gene Expression Omnibus (GEO)/NCBI database; the reports followed NIH research ethics guidelines [8], [9].

### Isolation of CD34+ HSC and liver progenitor cells from human fetal liver

Human liver progenitor cells containing hepatoblasts (Hep) and CD34+ hematopoietic stem cells (HSC) were isolated from 15–19 weeks old human fetal liver tissue (Advanced Bioscience Resources) essentially as described [56], [57], [58]. To separate progenitor liver cells from non-parenchymal cells (including HSC), the fetal liver cells were centrifuged at low speed three times (5 minutes, 18×*g*). Liver progenitor cells were collected in the pellet. The supernatant was centrifuged at 469×g for 5 min to collect the non-parenchyma mononuclear cells. CD34+ cells were isolated by magnetic-activated cell sorting (MACS), and the purity of CD34+ HSCs was greater than 95%. Cell viability, measured using Guava Easycyte -with Viacount staining (Millipore), generally exceeded 90%.

### Construction of A2/NSG/Fas-hu mice with human immune and liver cells

CD34+ HSCs (0.5–1×10^6^) and Hep (liver) progenitor cells (0.5–1×10^6^) from the same donor liver were co-injected into the liver of 1 to 2 days old newborn A2/NSG mice, previously irradiated at 200 rad. Additionally, fetal thymus tissue from the same donor was also transplanted when available. Animals were injected 3–5 times via ip with Jo2 antibody/PBS at 0.1–0.15 mg/kg body weight (BD Pharmingen) every 4–5 days at approximately 3–4 weeks post transplant of human cells [59]. At 12–16 weeks post-transplant with HSC+Hep cells, Transplanted mice were bled to determine human leukocyte (hCD45+) reconstitution by FACS and human albumin concentration in the blood by ELISA (Bethyl laboratories). All experiments using live rodents conformed to governmental and institutional guidelines.

### HBV infection of humanized mice

HBV clinical isolates were obtained from patients with chronic HBV infection (patient # 1 – HBV #1 and patient # 2 – HBV #2). A2/NSG-humanized mice or non-humanized control mice were inoculated *iv* with 50–75 ul of clinical isolates of HBV (1×10^3^–1×10^7^ genome equivalent copies) plus or minus anti-HBsAg ScFv neutralizing antibody (NAb) mixture (40 ug) per mouse or vehicle control (PBS). For in vivo neutralization assay, virus plus or minus monoclonal antibodies mixture was incubated at 25 degrees (room temperature) for 1 hour prior to inoculation.

### Blood and tissue analysis of A2/NSG/Fas-humanized mice

At different times after infection, blood was collected and HBsAg levels were measured using in house ELISA consisting of anti-HBsAg ScFv monoclonal antibodies mixture and commercial reagents (Alpha Diagnostics). HBV serum genome was detected at termination using real-time PCR [60]. Serum cytokine levels were measure using a multiplex human cytokine array and following manufacturer's recommended procedures (Luminex, Millipore). Liver fibrosis serum biomarkers, gamma-glutamyl transpeptidase (GGT) was measure using MaxDiscovery GGT enzymatic assay (Bioo Scientific Corporation) and hyaluronic acid (HA) was measured using hyaluronic acid ELISA kit (Echelon Biosciences) were measure following manufacturer's recommended procedures. At termination, liver tissue was immediately place in RNAlater (Qiagen) or fixed in 10% formalin. RNAlater was removed from tissue and samples were stored in −80 C. RNA was isolated from tissue following manufacturer's recommended procedures and qPCR was performed using species-specific published [18], [61] or NCBI primer blast generated primers and the SYBR Green method, following manufacturer's recommended procedures (Thermo Scientific). Paraffin embedded fixed liver sections were stained with hematoxylin and eosin (H&E), sirius red/fast green (fibrosis), Masson's trichrome (fibrosis) or with antibodies: anti-human GFAP (1∶250; Abcam), anti-human Collagen 1 (1∶250; Abcam), anti-mouse Collagen 1 (1∶250; Abcam), anti-human α-smooth muscle actin (1∶75; Dako), anti-human CD45 (1∶2, Dako), anti-human CD3 (1∶250; Dako), anti-human CD68 (1∶250; Dako), anti-human albumin (1∶250; Dako), anti-Hep Par1 (Dako), anti-HBcAg (1∶100; Zeta Corp). anti-HBsAg (1∶100; Thermo Scientific). Immunoreactivity was determined by incubation with DAB substrate (Pierce) or Vulcan red (Dako), and counterstained with hematoxilin [56], [58]. Liver histological activity was determined using the knodell score, which examines liver necrosis, degeneration, inflammation and fibrosis [62], [63].

### T cell immune responses in mock or HBV-infected A2/NSG/Fas-hu mice

Spleens, mesenteric lymph nodes and liver were isolated from individual animals and 0.5×10^6^ human spleen plus mesenteric lymph node or liver leukocytes were stimulated for 20 hours with phytohemagglutinin (PHA) or HBV antigens [HLA2 core (18–27), envelope (183–191, 185–194, 172–181), polymerase (573–581) peptides (ProImmune) +/− recombinant HBc and HBs (ProSpec) at 10 ug/ml each]+1 ug/ml anti-CD28 mAb (Invitrogen) in IMDM+10% FBS media (Gibco). The cells were then cultured for 14 days with fresh media replaced every two days (IMDM, 10% FBS, 10 U/mL human IL-2 and 125 ng/mL IL-7; human T cell expansion was examined by FACs (Guava, Millipore) using CD45, CD3 and live cell marker. Additionally, cells were stain for HBV core (18–27) or HBV envelope (183–191) pentamer+ human CD8+ T cells (ProImmune). PHA expanded cells were re-stimulated with Phorbol 12-myristate 13-acetate (PMA) plus ionomycin for approximately 6 hours and cytokine secretion was block with brefeldin A; intracellular cytokine levels were examined using FACs.

### Human patient samples and analyses

Chronic HBV Infection and associated liver diseases: Fifty-two chronic HBV (CHB) patients and 22 liver cancer (LC) patients were recruited for this study. All patients were diagnosed according to our previously described criteria [64], [65] and had not received any antiviral therapies or immunosuppressive drugs within six months before sampling. Sixteen age- and sex-matched healthy individuals were enrolled as controls (HC). Individuals with concurrent HCV, HDV or HIV infections, autoimmune liver diseases or alcoholic liver disease were excluded.

Acute HBV - induced accelerated liver disease and failure: Gene expression analysis was performed on publicly available microarray dataset from patients with acute HBV-associated liver failure (n = 4 patients, 17 different liver specimens approximately evenly taken from the 4 livers) and match healthy control liver donors (n = 10); acute HBV- induced liver failure patients were previously healthy and had no signs of chronic liver disease [8]. Microarray dataset (GEO accession: GSE49656; [8]) was analyzed using GEOR (NCBI Software).

### Human macrophage culture

PBMC were isolated from the buffy coats of healthy HIV-1/HBV/HCV sero/qPCR negative blood donors by Ficoll-paque density gradient centrifugation. The cells were then washed, resuspended in RPMI containing pen/strep (1%), glutamine (1%), heat-inactivated FBS (10%), and seeded into tissue culture plates. Non-adherent cells, mostly T lymphocytes, were removed by gentle pipette aspiration after 1.5 h of incubation at 37°C in a humidified atmosphere containing 5% CO_2_. An equal volume of fresh complete medium was then added to each flask and attached cells at approximately 70–80% confluency were cultivated for 6 additional days at 37°C in 5% CO_2_ in presence of either rHuGM-CSF (100 ng/ml) (M1) or rHuM-CSF (100 ng/ml) (M2) for differentiation into polarized M1 or M2 - monocyte derived macrophages. These polarized macrophages (≥95% CD14^+^) were then stimulated for two days with either IFN-γ (50 ng/ml) and LPS (10 ng/ml) or IL-4 (20 ng/ml) to obtain activated M1 macrophages or activated M2 macrophages, respectively. Polarized or activated primary monocyte derived macrophages were washed and treated with medium containing HBV (HBV positive HepG2 cell line - HepG2.2.15 supernatant derived at MOI = 10) or Mock (HBV negative HepG2 cell line - HepG2 supernatant derived) for 6 days; M1 and M2 macrophage activation were examined using cytokine analysis (BD Biosciences) and qPCR (Invitrogen) following manufactures' recommended procedures.

### Statistical analysis

We used unpaired two-tailed Student's t-tests or ANOVA for all comparisons. p<0.05 is considered significant. All data are reported as means ± standard error.

## Supporting Information

Figure S1 **Anti-mouse Fas activating antibody exhibits murine-specific affinity and hepatotoxicity.** (**A**) Human hepatocyte cell line (HepG2) was culture in DMEM based complete medium and stained with PE-conjugated anti-human CD95 antibody (clone DX2, eBioscience) or PE-conjugated anti-mouse CD95 antibody (Jo2 antibody) (BD Pharmingen) and analyzed by FACs; Jo2 exhibits species-specific affinity for murine CD95. (**B**) Human fetal liver progenitor cells (7e5 cells per well) were cultured in 12 well plates and treated with Jo2 (500 ng/mL) or vehicle (PBS) for 3 days and cell death was examined using both FACs based viability staining assay. Treatment of fetal liver progenitor cells culture with Jo2 does not induce apoptosis. (n = 3 per treatment group) (**C**) For studies examining Fas activating antibody in vivo hepatotoxic activity, A2/NSG immunodeficient mice were injected once via ip with Jo2 at sub-lethal doses or vehicle (PBS) and ALT levels was measure 20 hours post treatment. Jo2 induces dose dependent murine liver damage in A2/NSG immunodeficient mice (n = 2 per treatment group). p<0.05 is considered significant. All data are reported as means ± standard error.
(PDF)

Figure S2 **Human immune reconstitution in A2/NSG/HSC-Hep Fas mice.** Leukocytes from indicated tissue were isolated and stain for various human immune lineages including T cells (hCD45+ hCD3+), B cells (hCD45+ hCD19+), monocytes/macrophages (hCD45+ hCD3− hCD19− hCD56− hHLADR+ hCD14^high^ hCD11C^high^), myeloid dendritic cells (hCD45+ hCD3− hCD19− hCD56− hHLADR+ hCD14^low^ hCD11C^high^) and plasmacytoid dendritic cells (hCD45+ hCD3− hCD19− hCD56− hHLADR+ hCD123^high^ hCD4^high^).
(PDF)

Figure S3 **HBV genome in the liver of humanized mice.** Extrachromosomal DNA was isolated from liver samples, and HBV DNA was quantified in mock (lanes 1–3) or HBV infected (lanes 4–8) humanized mice using real-time PCR (**A**) and gel electrophoresis analysis (**B**). HBV plasmid DNA from hydrodynamically transfected mice was used as positive control of qPCR and gel analysis (data not shown).
(PDF)

Figure S4 **HBV infection induces liver fibrosis/scarring in humanized mice.** Representative gross liver morphology of HBV-infected and mock-infected mice. HBV infection of humanized animals was associated with gross liver pathology with prominent scarring visible on the tissue.
(PDF)

Figure S5 **HBV infection primes human T cells in humanized mice.** Representative FACs analysis of Th1 associated double positive cytokine secretion in vehicle or PMA plus ionomycin re-stimulated PHA expanded T cells.
(PDF)

Figure S6 **Liver specific localization of human M2-like macrophages in HBV infected humanized mice.** Immunohistochemical analysis of M2 macrophage (CD68+, CD163+) levels in the spleen and liver of the same HBV-infected animal. Black arrows serve as a marker to denote the same region.
(PDF)

Figure S7 **Chronic HBV infection–induced liver fibrosis is associated with M2-like macrophages in humanized mice.** (**A–B**) Livers from representative HBV infected humanized mice (HBV), mock (Mock) inoculated humanized mice and HBV inoculated non-humanized (NTP-HBV) control mice were stained for M2-like (CD163+, brown regions) macrophages and liver fibrosis (MT, blue regions) and slides were scanned (**A**); additionally an enlarged HBV infected liver is shown (**B**), black arrows serve as a marker to denote the same region. (**C**) Quantitative analysis of CD163+ macrophages (brown color) and liver fibrosis (collagen deposition-blue color) in the indicated livers.
(PDF)

Figure S8 **Acute HBV infection–induced accelerated liver fibrosis and damage is associated with the induction of M2-like macrophage gene expression profile in humans.** Relative log_2_ expression of macrophage (CD68), M2-like macrophage (IL10RA, Dectin-1, CD163, CD163L1, MRC1(CD206), MRC2, AMAC1, IL10, B7-H4) and M1-like macrophage (TNFα, iNOS, IL12p40) markers in the livers of healthy control liver donors (n = 10) and human patients with acute HBV – induced liver failure (n = 17 from 4 patients).
(PDF)

Figure S9 **M2-like macrophage gene expression profile directly correlates with liver fibrosis and damage markers in Acute-HBV liver failure patients.** Regression/correlation analysis of relative log_2_ expression (RE) of macrophage (CD68) marker and M2-like macrophage (CD163, AMAC1) or M1-like macrophage (TNFα, iNOS) markers in the livers of human patients with acute HBV – induced liver failure show elevated levels of M2-like macrophage directly correlate with macrophage levels. Additionally, regression/correlation analysis of relative log_2_ expression of M2-like macrophage (CD163) marker and tissue fibrosis (COL1A1, TIMP1) or damage (HMGB1, S100A9) markers show M2-like macrophage levels correlate with liver fibrosis and damage levels.
(PDF)

Table S1 **Chronic HBV infection induces liver disease in the humanized mouse model.** Animal information and associated human reconstitution level, infection status, and liver disease score.
(PDF)

Table S2 **Chronic HBV–induced liver disease and immune impairment is associated with M2-like macrophages in the humanized mouse model.** Animal information, human reconstitution status, infection status, lymphoid and liver immune response, liver disease stage/score and associated liver M2-like macrophage levels.
(PDF)
