# Correction: Hepatitis B Virus Infection and Immunopathogenesis in a Humanized Mouse Model: Induction of Human-Specific Liver Fibrosis and M2-Like Macrophages

**DOI:** 10.1371/journal.ppat.1004718

**Published:** 2015-03-17

**Authors:** The PLOS Pathogens Staff

In the Funding section, the grant from National Science and Technology Major Project (2013ZX10002008) is omitted.

The correct funding statement should read:

This work was supported in part by grants from UNC UCRF innovation grant, from NIH: UNC SPORE grants; AI076142, AA018009 (LS), and UNC Lineberger Comprehensive Cancer Center and UNC Infectious Disease Pathogenesis Postdoctoral Training Grants (MTB); from Chinese MOST 2009CB522507, MOH 2012ZX10004503-007 (LZ) and from National Science and Technology Major Project 2013ZX10002008(ZT, JN, LS). The funders had no role in study design, data collection and analysis, decision to publish, or preparation of the manuscript.
