# Supplementary figures and images for: Hepatitis B Virus Infection and Immunopathogenesis in a Humanized Mouse Model: Induction of Human-Specific Liver Fibrosis and M2-Like Macrophages
Source: PLoS Pathog. 2014 Mar 20;10(3):e1004032. doi: 10.1371/journal.ppat.1004032 (PMC3961374; doi:10.1371/journal.ppat.1004032)

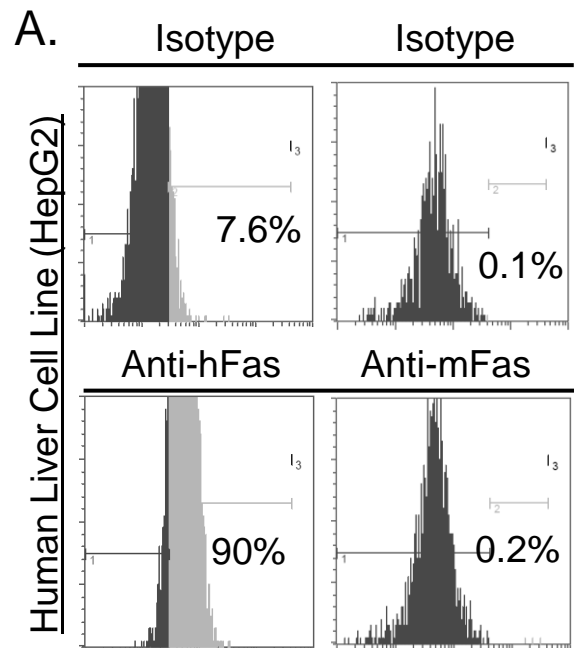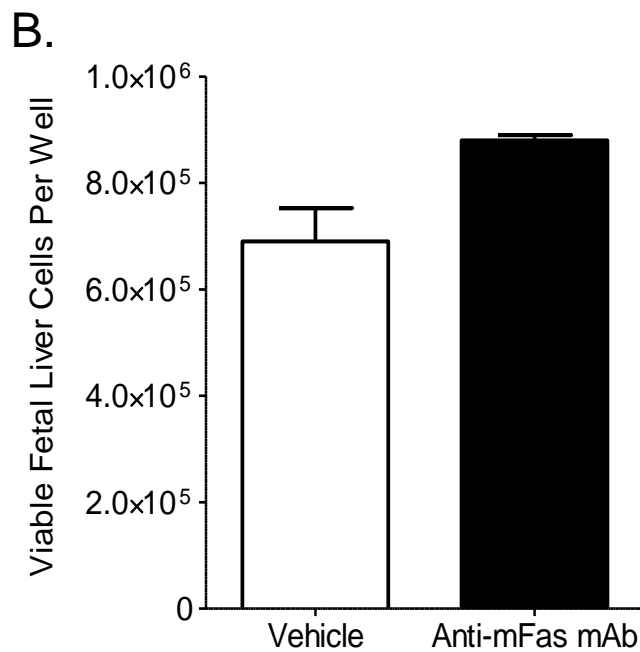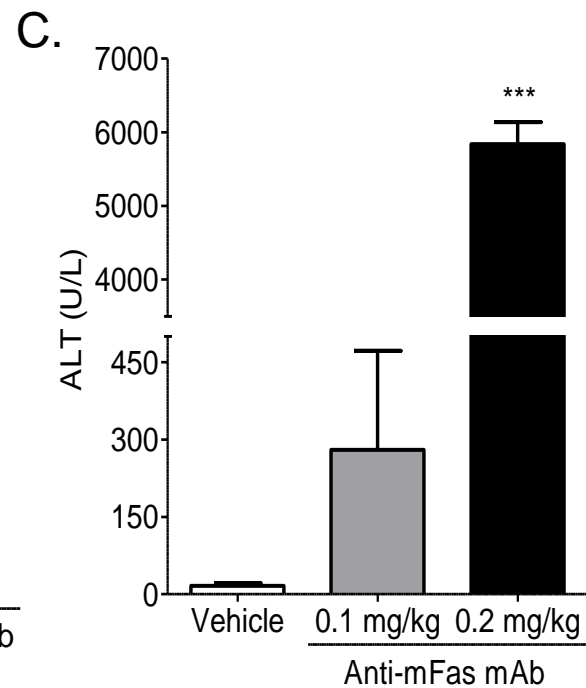

Supplement: Figure S1 — Anti-mouse Fas activating antibody exhibits murine-specific affinity and hepatotoxicity. (A) Human hepatocyte cell line (HepG2) was culture in DMEM based complete medium and stained with PE-conjugated anti-human CD95 antibody (clone DX2, eBioscience) or PE-conjugated anti-mouse CD95 antibody (Jo2 antibody) (BD Pharmingen) and analyzed by FACs; Jo2 exhibits species-specific affinity for murine CD95. (B) Human fetal liver progenitor cells (7e5 cells per well) were cultured in 12 well plates and treated with Jo2 (500 ng/mL) or vehicle (PBS) for 3 days and cell death was examined using both FACs based viability staining assay. Treatment of fetal liver progenitor cells culture with Jo2 does not induce apoptosis. (n = 3 per treatment group) (C) For studies examining Fas activating antibody in vivo hepatotoxic activity, A2/NSG immunodeficient mice were injected once via ip with Jo2 at sub-lethal doses or vehicle (PBS) and ALT levels was measure 20 hours post treatment. Jo2 induces dose dependent murine liver damage in A2/NSG immunodeficient mice (n = 2 per treatment group). p<0.05 is considered significant. All data are reported as means ± standard error. (PDF) [file ppat.1004032.s001.pdf]

A2/NSG - Fas HSC/Hep hu Mice - Tissue Leukocytes

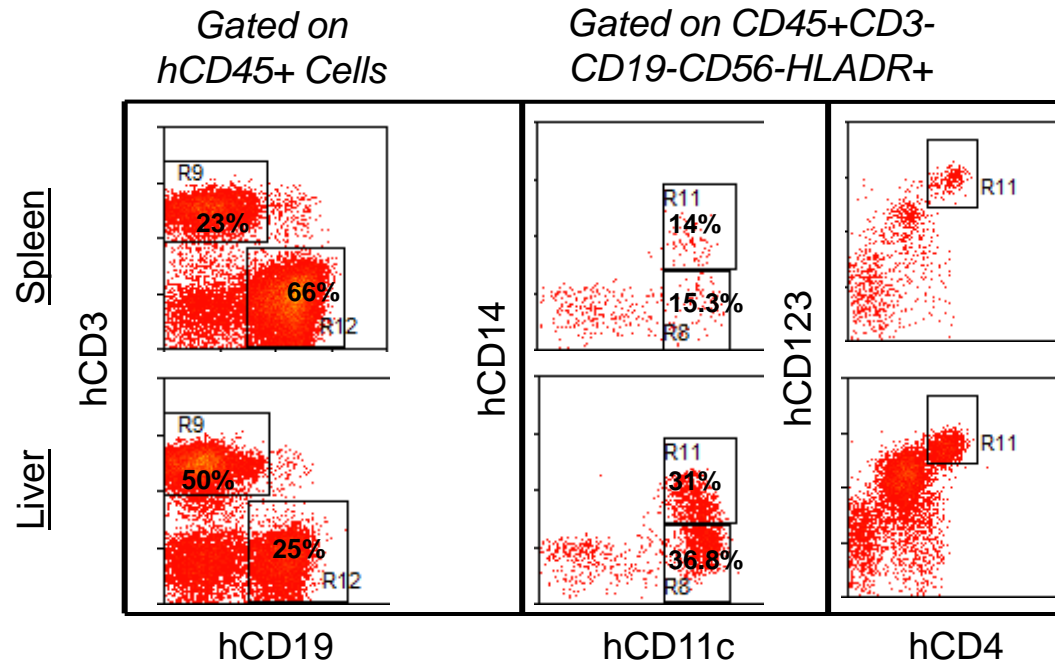

Supplement: Figure S2 — Human immune reconstitution in A2/NSG/HSC-Hep Fas mice. Leukocytes from indicated tissue were isolated and stain for various human immune lineages including T cells (hCD45+ hCD3+), B cells (hCD45+ hCD19+), monocytes/macrophages (hCD45+ hCD3− hCD19− hCD56− hHLADR+ hCD14high hCD11Chigh), myeloid dendritic cells (hCD45+ hCD3− hCD19− hCD56− hHLADR+ hCD14low hCD11Chigh) and plasmacytoid dendritic cells (hCD45+ hCD3− hCD19− hCD56− hHLADR+ hCD123high hCD4high). (PDF) [file ppat.1004032.s002.pdf]

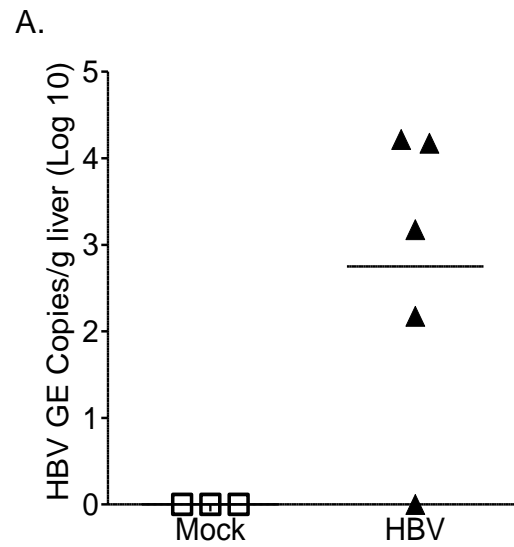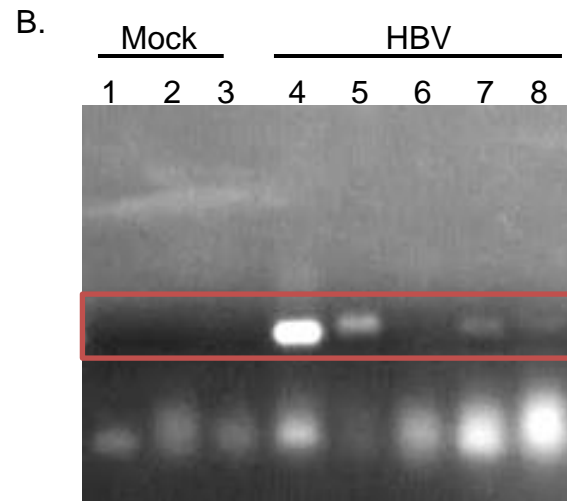

Supplement: Figure S3 — HBV genome in the liver of humanized mice. Extrachromosomal DNA was isolated from liver samples, and HBV DNA was quantified in mock (lanes 1–3) or HBV infected (lanes 4–8) humanized mice using real-time PCR (A) and gel electrophoresis analysis (B). HBV plasmid DNA from hydrodynamically transfected mice was used as positive control of qPCR and gel analysis (data not shown). (PDF) [file ppat.1004032.s003.pdf]

Mock

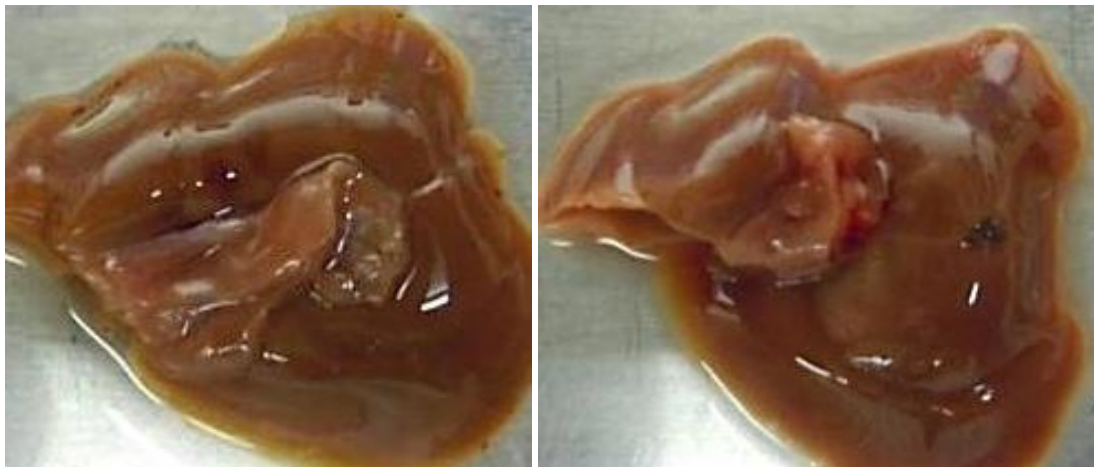

HBV

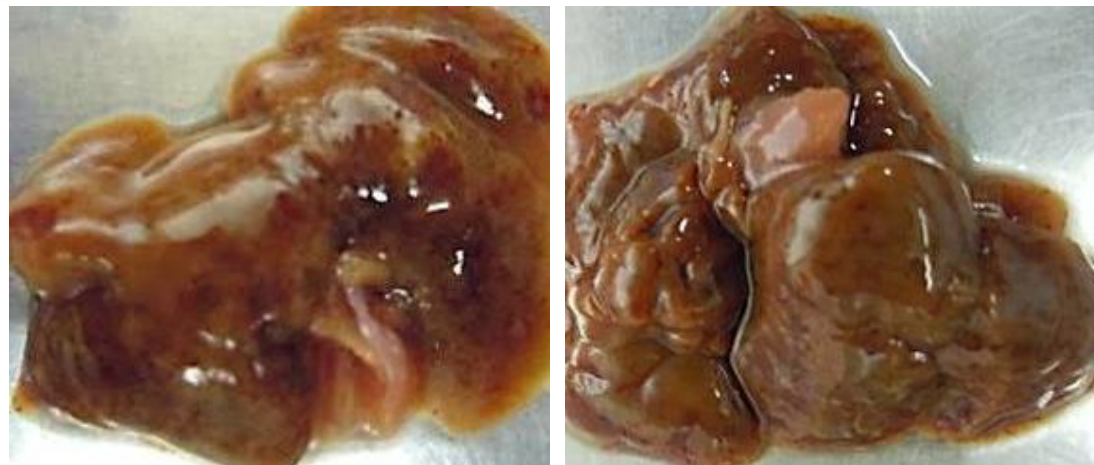

Supplement: Figure S4 — HBV infection induces liver fibrosis/scarring in humanized mice. Representative gross liver morphology of HBV-infected and mock-infected mice. HBV infection of humanized animals was associated with gross liver pathology with prominent scarring visible on the tissue. (PDF) [file ppat.1004032.s004.pdf]

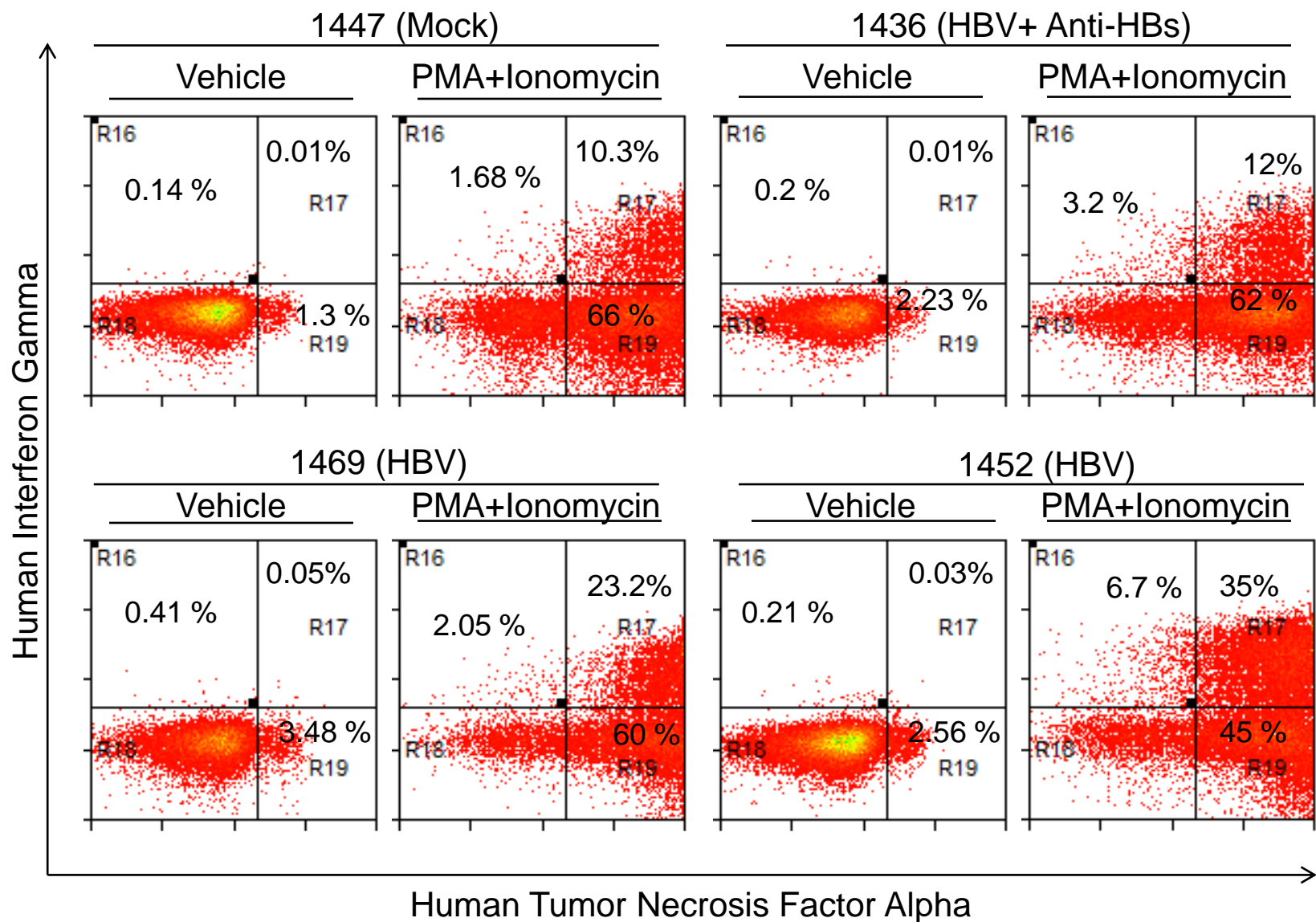

Supplement: Figure S5 — HBV infection primes human T cells in humanized mice. Representative FACs analysis of Th1 associated double positive cytokine secretion in vehicle or PMA plus ionomycin re-stimulated PHA expanded T cells. (PDF) [file ppat.1004032.s005.pdf]

HBV Infected Humanized Mice

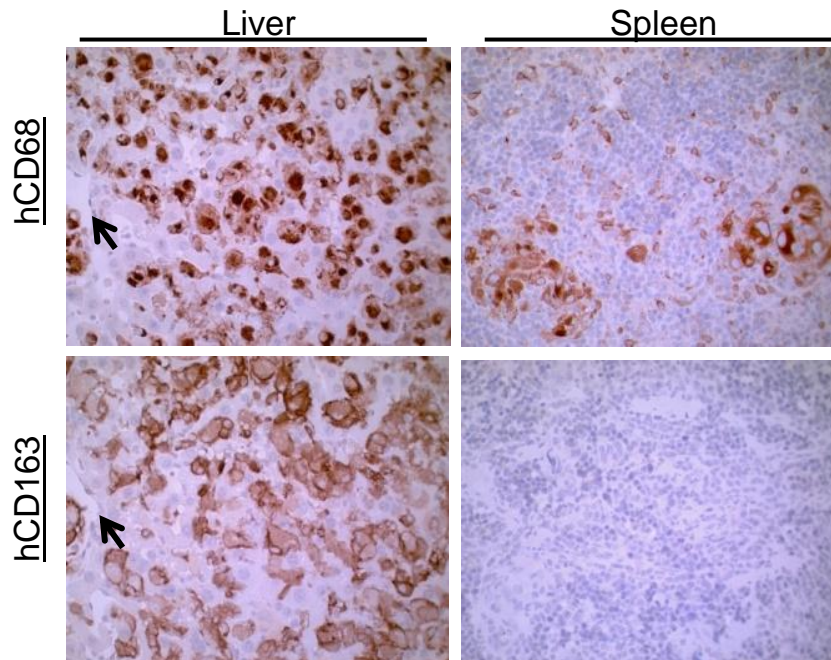

Supplement: Figure S6 — Liver specific localization of human M2-like macrophages in HBV infected humanized mice. Immunohistochemical analysis of M2 macrophage (CD68+, CD163+) levels in the spleen and liver of the same HBV-infected animal. Black arrows serve as a marker to denote the same region. (PDF) [file ppat.1004032.s006.pdf]

A.

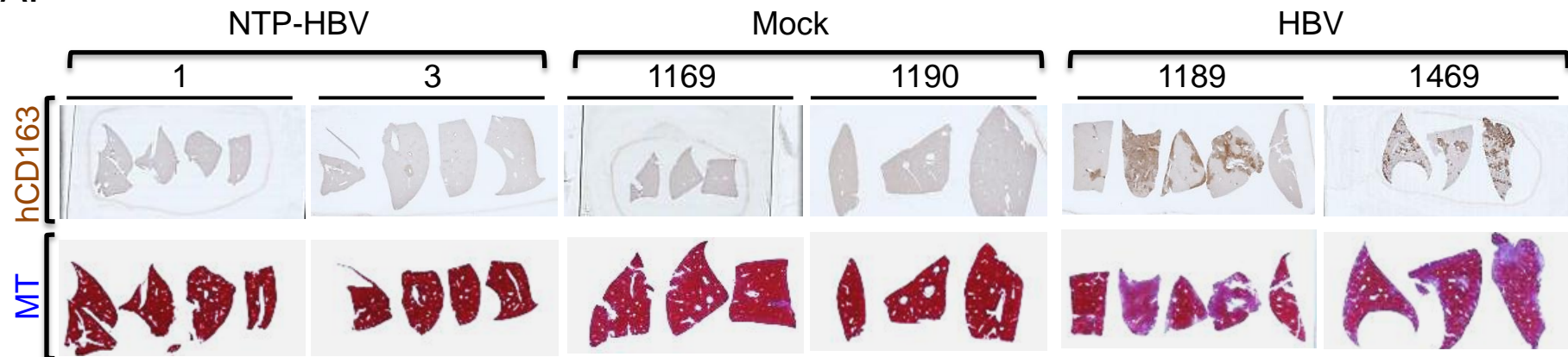

B.

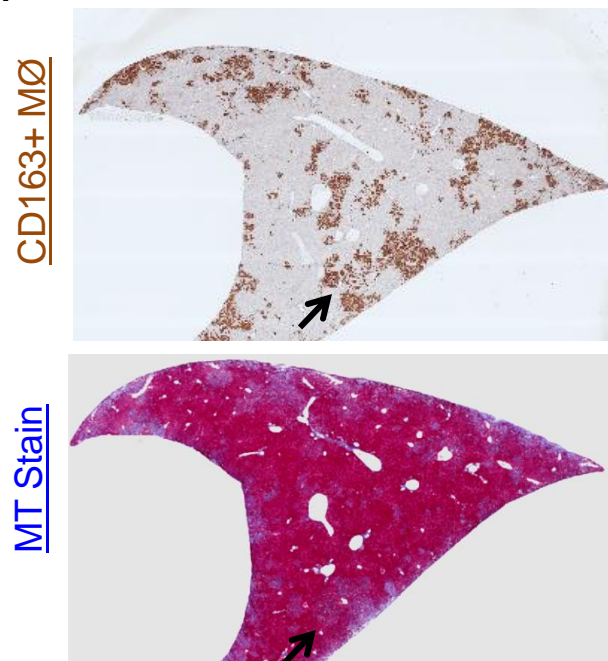

C.

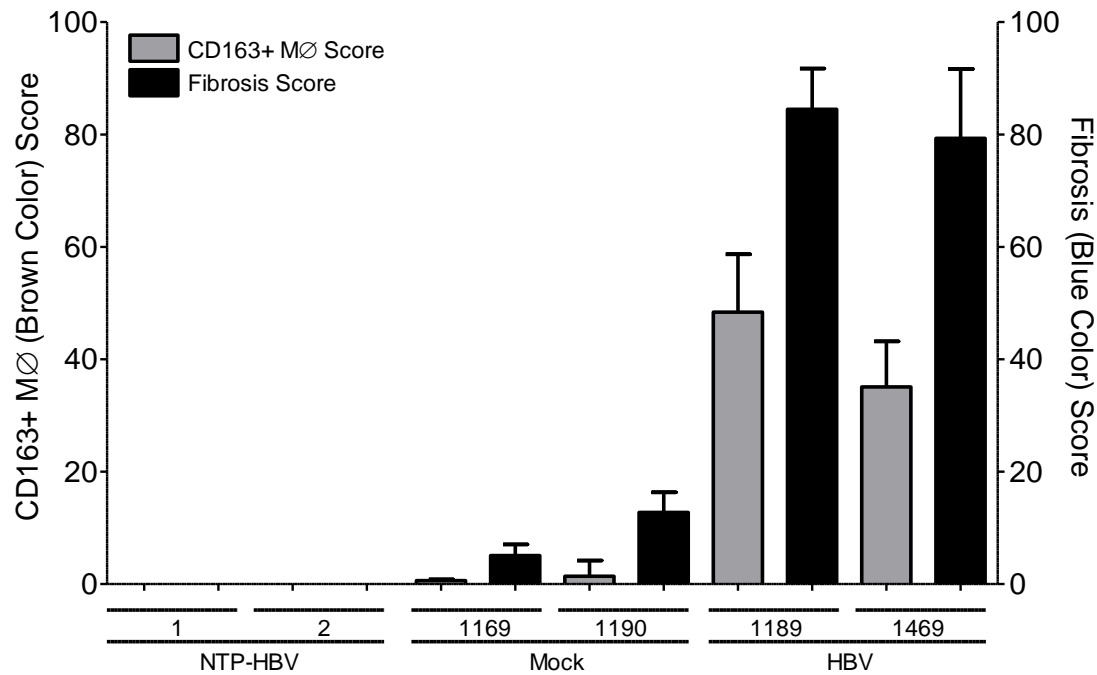

Supplement: Figure S7 — Chronic HBV infection–induced liver fibrosis is associated with M2-like macrophages in humanized mice. (A–B) Livers from representative HBV infected humanized mice (HBV), mock (Mock) inoculated humanized mice and HBV inoculated non-humanized (NTP-HBV) control mice were stained for M2-like (CD163+, brown regions) macrophages and liver fibrosis (MT, blue regions) and slides were scanned (A); additionally an enlarged HBV infected liver is shown (B), black arrows serve as a marker to denote the same region. (C) Quantitative analysis of CD163+ macrophages (brown color) and liver fibrosis (collagen deposition-blue color) in the indicated livers. (PDF) [file ppat.1004032.s007.pdf]

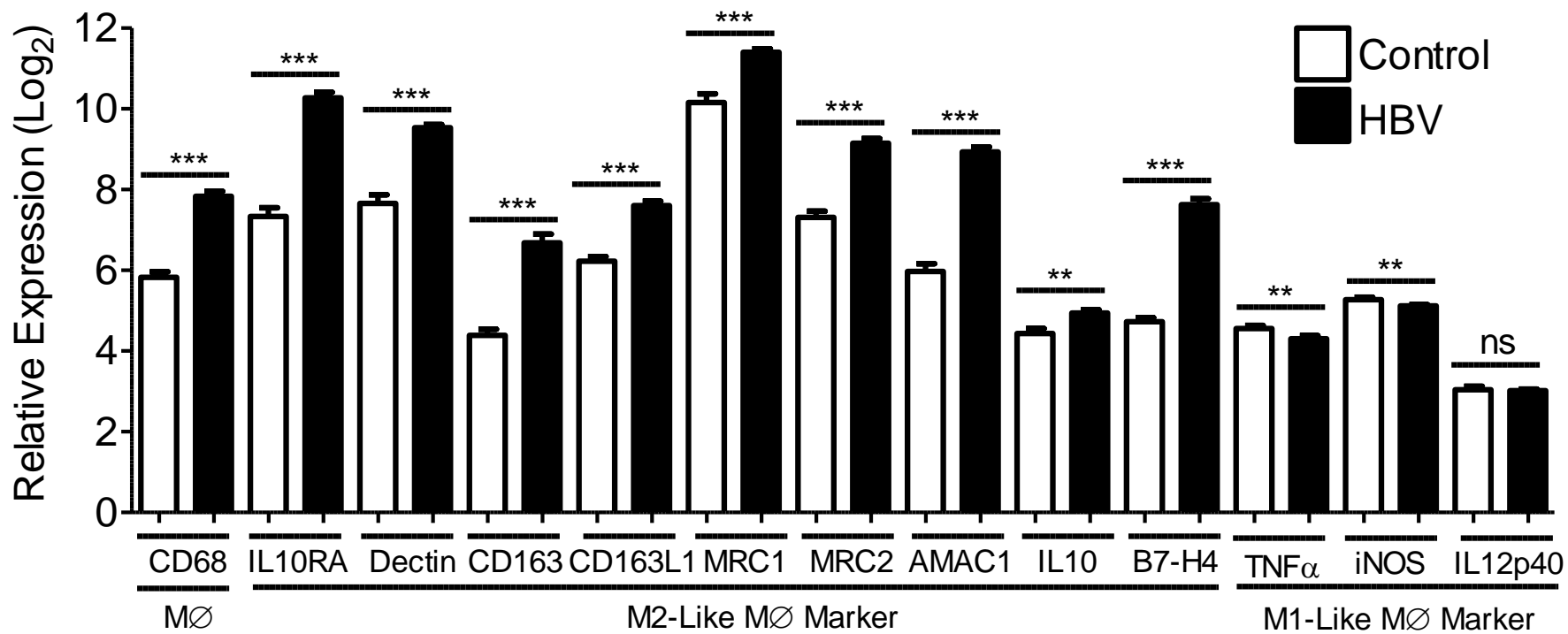

Supplement: Figure S8 — Acute HBV infection–induced accelerated liver fibrosis and damage is associated with the induction of M2-like macrophage gene expression profile in humans. Relative log2 expression of macrophage (CD68), M2-like macrophage (IL10RA, Dectin-1, CD163, CD163L1, MRC1(CD206), MRC2, AMAC1, IL10, B7-H4) and M1-like macrophage (TNFα, iNOS, IL12p40) markers in the livers of healthy control liver donors (n = 10) and human patients with acute HBV – induced liver failure (n = 17 from 4 patients). (PDF) [file ppat.1004032.s008.pdf]

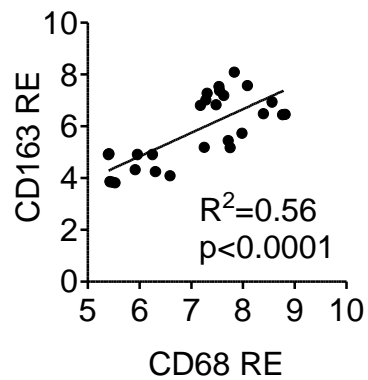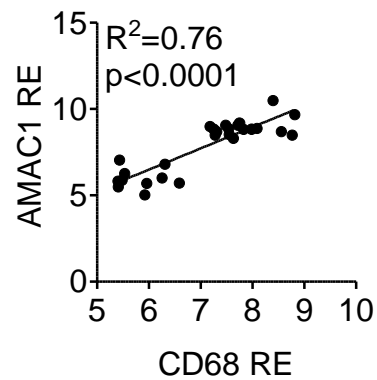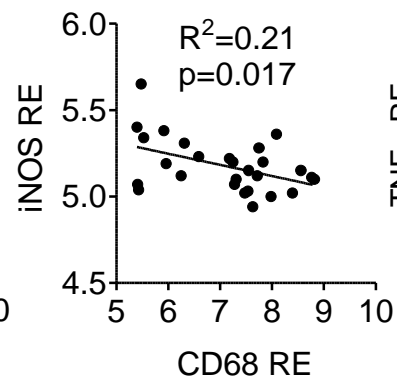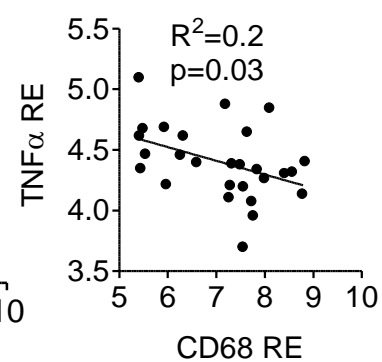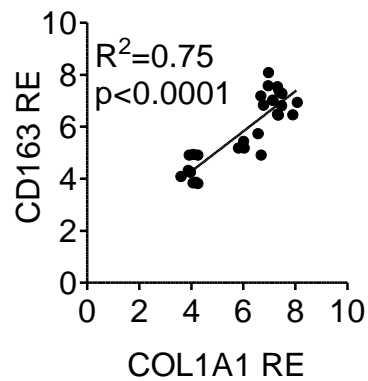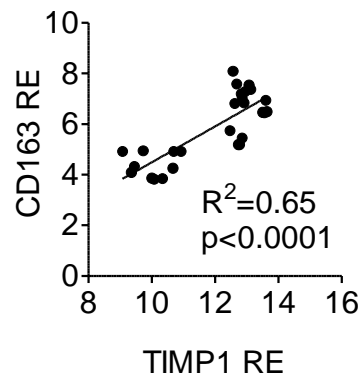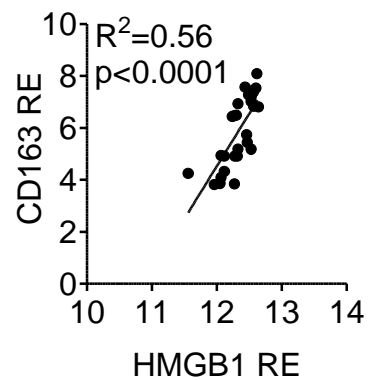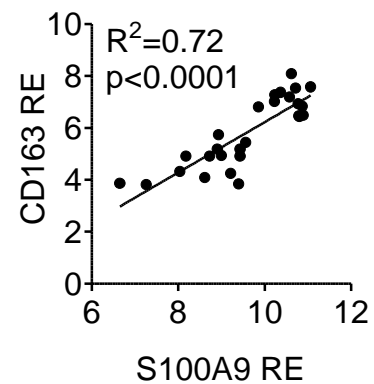

Supplement: Figure S9 — M2-like macrophage gene expression profile directly correlates with liver fibrosis and damage markers in Acute-HBV liver failure patients. Regression/correlation analysis of relative log2 expression (RE) of macrophage (CD68) marker and M2-like macrophage (CD163, AMAC1) or M1-like macrophage (TNFα, iNOS) markers in the livers of human patients with acute HBV – induced liver failure show elevated levels of M2-like macrophage directly correlate with macrophage levels. Additionally, regression/correlation analysis of relative log2 expression of M2-like macrophage (CD163) marker and tissue fibrosis (COL1A1, TIMP1) or damage (HMGB1, S100A9) markers show M2-like macrophage levels correlate with liver fibrosis and damage levels. (PDF) [file ppat.1004032.s009.pdf]
