# Supplementary material for: Hepatitis B Virus Infection and Immunopathogenesis in a Humanized Mouse Model: Induction of Human-Specific Liver Fibrosis and M2-Like Macrophages
Source: PLoS Pathog. 2014 Mar 20;10(3):e1004032. doi: 10.1371/journal.ppat.1004032 (PMC3961374; doi:10.1371/journal.ppat.1004032)
Supplement: Table S1 — Chronic HBV infection induces liver disease in the humanized mouse model. Animal information and associated human reconstitution level, infection status, and liver disease score. (PDF) [file ppat.1004032.s010.pdf]

Chronic HBV infection induces liver disease in the humanized mouse model

ID	Model	%hCD45	Serum hAlb (ng/ml)	Inoculum	WPI	Serum HBs (ng/ml)	Liver HBc	Liver HBs	Knodell Score
1168	Hu-mice	87.1	40	Mock	14	-	-	-	0
1169	Hu-mice	88.9	46	Mock	14	-	-	-	0
1170	Hu-mice	95.2	52	Mock	14	-	-	-	0
1190	Hu-mice	12.8	22	Mock	10	-	-	-	1
1447	Hu-mice	92	67	Mock	12	-	-	-	0
1448	Hu-mice	3	26	Mock	16	-	-	-	0
1434	Hu-mice	89	51	HBV#2+Nab	16	-	-	-	1
1435	Hu-mice	91	30	HBV#2+Nab	16	-	-	-	0
1436	Hu-mice	84	91	HBV#2+Nab	12	-	-	-	0
1189	Hu-mice	70.8	72	HBV#1	14	9.7	+	+	16
1191	Hu-mice	78.4	106	HBV#1	6	6.25	+	+	14
1192	Hu-mice	35.4	14	HBV#1	10	3.29	NA	NA	NA
1193	Hu-mice	93.2	118	HBV#1	13	7.3	+	+	16
1441	Hu-mice	20	146	HBV#2	11	8.8	NA	NA	NA
1442	Hu-mice	66	189	HBV#2	16	6.3	+	+	6
1450	Hu-mice	94	300	HBV#2	16	-	-	-	0
1452	Hu-mice	85	84	HBV#2	12	8.2	+	+	10
1469	Hu-mice	64	52	HBV#2	14	4.75	+	+	17
1476	Hu-mice	69	26	HBV#2	14	-	-	-	0
1477	Hu-mice	79	97	HBV#2	14	4.84	+	+	14
1	Non-hu mice	0	0	HBV#1	16	-	-	-	0
2	Non-hu mice	0	0	HBV#1	16	-	-	-	0
3	Non-hu mice	0	0	HBV#1	16	-	-	-	0
4	Non-hu mice	0	0	HBV#2	16	-	-	-	0
5	Non-hu mice	0	0	HBV#2	16	-	-	-	0
6	Non-hu mice	0	0	HBV#2	16	-	-	-	0

Notes: ID=Identification; Hu-mice=A2/NSG-hu HSC/Hep mouse; Non-hu mice=Non transplanted A2/NSG mouse; %hCD45=Human immune cells reconstitution levels relative to mouse immune cells; Serum hAlb=Serum human albumin levels; Inoculum=Mock (Vehicle; PBS), HBV#1 (HBV positive serum from patient #1), HBV#2 (HBV positive serum from patient #2), HBV#2+NAb (HBV positive serum from patient #1 incubated with neutralizing antibody prior to inoculation), WPI=Weeks post inoculation and time of death; NA=Not applicable (Animal was found dead); Serum HBs=Not detected (-) or concentration; Liver HBs=Not detected (-) or detected (+); Liver HBc=Not detected (-) or detected (+); Knodell=Liver histological activity score.
