# Supplementary material for: Hepatitis B Virus Infection and Immunopathogenesis in a Humanized Mouse Model: Induction of Human-Specific Liver Fibrosis and M2-Like Macrophages
Source: PLoS Pathog. 2014 Mar 20;10(3):e1004032. doi: 10.1371/journal.ppat.1004032 (PMC3961374; doi:10.1371/journal.ppat.1004032)
Supplement: Table S2 — Chronic HBV–induced liver disease and immune impairment is associated with M2-like macrophages in the humanized mouse model. Animal information, human reconstitution status, infection status, lymphoid and liver immune response, liver disease stage/score and associated liver M2-like macrophage levels. (PDF) [file ppat.1004032.s011.pdf]

Chronic HBV – induced liver disease and immune impairment is associated with "M2-like" macrophages in the humanized mouse model.

ID	Model	Inoculum	Chronic HBV	Lymphoid T cells Immune Response	Liver T cells Immune Response	Liver Disease	Liver M2 MØ
1168	Hu-mice	Mock	-	-	NA*	-	-
1169	Hu-mice	Mock	-	NA	NA	-	-
1170	Hu-mice	Mock	-	-	NA*	-	-
1190	Hu-mice	Mock	-	NA	NA	-	-
1447	Hu-mice	Mock	-	-	-	-	-
1448	Hu-mice	Mock	-	NA	NA	-	-
1434	Hu-mice	HBV+Nab	-	-	-	-	-
1435	Hu-mice	HBV+Nab	-	NA	NA	-	-
1436	Hu-mice	HBV+Nab	-	NA	NA	-	-
1189	Hu-mice	HBV	+	+	-	+++	+++
1191	Hu-mice	HBV	+	+	NA	+++	+++
1193	Hu-mice	HBV	+	NA	NA	+++	+++
1442	Hu-mice	HBV	+	NA	NA	+	+
1450	Hu-mice	HBV	-	NA	NA	-	-
1452	Hu-mice	HBV	+	+	-	++	++
1469	Hu-mice	HBV	+	+	-	+++	+++
1476	Hu-mice	HBV	-	+	+	-	-
1477	Hu-mice	HBV	+	+	-/+	+++	+++
1	Non-hu mice	HBV	-	NA	NA	-	-
2	Non-hu mice	HBV	-	NA	NA	-	-
3	Non-hu mice	HBV	-	NA	NA	-	-
4	Non-hu mice	HBV	-	NA	NA	-	-
5	Non-hu mice	HBV	-	NA	NA	-	-
6	Non-hu mice	HBV	-	NA	NA	-	-

Notes: ID=Identification; Hu-mice=A2/NSG-hu HSC/Hep mouse; Non-hu mice=Non transplanted A2/NSG mouse; Inoculum=Mock (Vehicle; PBS), HBV#1 (HBV positive serum from patient #1), HBV#2 (HBV positive serum from patient #2), HBV#2+NAb (HBV positive serum from patient #1 incubated with neutralizing antibody prior to inoculation); Chronic HBV= Persistent HBV infection markers (HBV genome and antigens) detected in animal; Lymphoid/Liver T cells immune response=Refractory (-), Intermediate response (-/+), Responsive (+); NA (Not applicable, animals not tested, NA* indicates animals had low number of T cells in the liver below the assay requirements); Liver disease=Relative liver disease score based on Knodell score, no liver disease (-), low grade liver disease (+), medium grade liver disease (+++), high grade liver disease (+++); Liver M2 MØ=Relative M2-like (hCD163+) macrophage levels, no M2 macrophages (-), low M2 macrophage level (+), intermediate M2 macrophage level (++), high M2 macrophage level (+++).
